# Supplementary material for: Impact of COVID-19 pandemic and anti-pandemic measures on tuberculosis, viral hepatitis, HIV/AIDS and malaria–A systematic review
Source: PLOS Glob Public Health. 2023 May 1;3(5):e0001018. doi: 10.1371/journal.pgph.0001018 (PMC10150989; doi:10.1371/journal.pgph.0001018)
Supplement: S1 Text — (PDF) [file pgph.0001018.s002.pdf]

S1 Supporting information to: Impact of COVID-19 pandemic and anti-pandemic measures on tuberculosis, viral hepatitis, HIV/AIDS and malaria – a systematic review

by B Kessel, T Heinsohn, J J Ott, J Wolff, M J Hassenstein, B Lange

A. Search strategy in databases

Database: PubMed, https://pubmed.ncbi.nlm.nih.gov/advanced/
Search 28.09.2020 and 13.01.2021:
((COVID-19) OR (SARS-Cov-2)) AND ((hepatitis) OR (tuberculosis) OR (HIV) OR (malaria))

("severe acute respiratory syndrome coronavirus 2"[Supplementary Concept] OR "severe acute respiratory syndrome coronavirus 2"[All Fields] OR "ncov"[All Fields] OR "2019 ncov"[All Fields] OR "covid 19"[All Fields] OR "sars cov 2"[All Fields] OR (("coronavirus"[All Fields] OR "cov"[All Fields]) AND 2019/11/01:3000/12/31[Date - Publication]) OR ("severe acute respiratory syndrome coronavirus 2"[Supplementary Concept] OR "severe acute respiratory syndrome coronavirus 2"[All Fields] OR "sars cov 2"[All Fields])) AND ("hepatitis"[MeSH Terms] OR "hepatitis"[All Fields] OR "hepatitides"[All Fields] OR "hepatitis a"[MeSH Terms] OR "hepatitis a"[All Fields] OR ("tuberculosi"[All Fields] OR "tuberculosis"[MeSH Terms] OR "tuberculosis"[All Fields] OR "tuberculoses"[All Fields] OR "tuberculosis s"[All Fields]) OR ("hiv"[MeSH Terms] OR "hiv"[All Fields]) OR ("malaria"[MeSH Terms] OR "malaria"[All Fields] OR "malarias"[All Fields] OR "malaria s"[All Fields] OR "malariae"[All Fields]))

Translations

COVID-19: "severe acute respiratory syndrome coronavirus 2"[Supplementary Concept] OR "severe acute respiratory syndrome coronavirus 2"[All Fields] OR "ncov"[All Fields] OR "2019-nCoV"[All Fields] OR "COVID-19"[All Fields] OR "SARS-CoV-2"[All Fields] OR ((coronavirus[All Fields] OR "cov"[All Fields]) AND 2019/11:3000[pdat])

SARS-Cov-2: "severe acute respiratory syndrome coronavirus 2"[Supplementary Concept] OR "severe acute respiratory syndrome coronavirus 2"[All Fields] OR "sars cov 2"[All Fields]

hepatitis: "hepatitis"[MeSH Terms] OR "hepatitis"[All Fields] OR "hepatitides"[All Fields] OR "hepatitis a"[MeSH Terms] OR "hepatitis a"[All Fields]

tuberculosis: "tuberculosi"[All Fields] OR "tuberculosis"[MeSH Terms] OR "tuberculosis"[All Fields] OR "tuberculoses"[All Fields] OR "tuberculosis's"[All Fields]

HIV: "hiv"[MeSH Terms] OR "hiv"[All Fields]

malaria: "malaria"[MeSH Terms] OR "malaria"[All Fields] OR "malarias"[All Fields] OR "malaria's"[All Fields] OR "malariae"[All Fields]

Search 13.01.2021: publication date from 2020/09/29

Database: preView (includes medRxiv, bioRxiv, ChemRxiv, ResearchSquare, arXiv, Preprints.org), https://preview.zbmed.de
Search 28.09.2020: tuberculosis OR HIV OR malaria OR hepatitis
Search 13.01.2021: tuberculosis OR HIV OR malaria OR hepatitis with
Publication date 2020/09/29
Publication date 2020/09/30
Publication date 2020/10
Publication date 2020/11
Publication date 2020/12
Publication date 2021/01

Database: Scopus, https://www.scopus.com/search/form.uri?display=advanced
Search 28.09.2020: TITLE-ABS-KEY(("SARS-CoV-2" OR "COVID-19") AND ("tuberculosis" OR "HIV" OR "hepatitis" OR "malaria"))
Search 13.01.2021: TITLE-ABS-KEY(("SARS-CoV-2" OR "COVID-19") AND ("tuberculosis" OR "HIV" OR "hepatitis" OR "malaria")) AND PUBDATETXT(„29 September 2020“ or „30 September 2020“ or „October 2020“ or „November 2020“ or „December 2020“ or „January 2021“)

Division of the identified records into groups according to TB/Malaria/Hepatitis/HIV: relevant papers for each disease were found using the search function in EndNote X9 and the following search terms in any field:
TB: tuberculosis OR tuberculoses OR TB

HIV: HIV OR HIV-1
Hepatitis: hepatitis OR hepatides OR HBV
Malaria: malaria OR malariae OR malarias

B. Search strategy for grey literature

S1 Table A Details of the grey literature search.

**Disease**	**Date of the search**	**Websites searched**
Viral hepatitis	Sep 30, 2020<br>Jan 28, 2021	The main medical societies in the field:<br>• The European Association for the Study of the Liver (EASL, www.easl.eu)<br>• The American Association for the Study of Liver Diseases (AASLD, www.aasld.org)<br>The largest umbrella-NGO in the area of hepatitis<br>• The World Hepatitis Alliance (WHA, www.worldhepatitisalliance.org)<br>Platform for international hepatitis news<br>• Hepatitis Voices(www.HepVoices.org)
Tuberculosis	Dec 2020	WHO (www.who.int), TB Union (theunion.org), The Global Fund (www.theglobalfund.org)
HIV/AIDS	Feb 1, 2021	WHO (www.who.int), UNAIDS (www.unaids.org), International AIDS Society (www.iasociety.org), Kaiser Family Foundation (www.kkf.org), European AIDS Treatment Group (www.eatg.org), Sensoa (www.sensoa.be), PEPFAR (www.state.gov/pepfar), The Global Fund (www.theglobalfund.org), AMPATH (www.amptahkenya.org), CAPRISA (www.caprisa.org), the virtual AIDS 2020 conference (http://programme.aids2020.org/)
Malaria	Jan 30, 2021	WHO (www.who.int), RBM Partnership to End Malaria (https://endmalaria.org/),The Global Fund (https://www.theglobalfund.org/en/)

C. Post-hoc amendments to the study protocol

For surveys, we accepted an implicit comparator, i.e. questions with wording “due to COVID-19”, or “under current travel restrictions”.

In the case of HIV, we adopted the following two additional exclusion criteria:

- small sample size: 20 or fewer respondents if not speaking on behalf of programmes or organizations,
- surveys in which a certain group of people was asked about the COVID-19 impact on another group (e.g. health care professionals were asked about the impact on patients).

We also did not consider survey questions regarding anticipated difficulties due to COVID-19, as opposed to experienced difficulties.

D. Details of the screening process and the “near-misses”

Tuberculosis

Three publications classified as near misses in the full-text screening. Two did not contain data related to COVID-19 [1, 2], one did not provide a comparator for the absolute impact [3].

HIV/AIDS

In full-text screening, we excluded 14 full-texts under the label: insufficient reporting/data. Here we give their details. Three [4-6] did not contain any precise quantifications in their statements. They reported endangered ART supplies in Indonesia[4], maintaining HIV therapy among people with substance use disorder in Ukraine[6] and cessation of community HIV testing in Birmingham, Alabama[5]. Four publications [7-10] did not contain information to which period exactly the reported data refer. They reported a decrease in PEP prescriptions (China)[8], interruptions in medication uptake(China)[9], no interruption in outpatient services and therapy withdrawal(Italy)[7] and self-reported problems to access HIV medication by 4% of respondents (Argentina)[10]. Further, four publications were based on surveys of and about individuals with 20 and fewer respondents, reporting no interruptions to HIV care(USA) [11] or no interruptions to access to ART(USA) [12], a decrease in PrEP usage(USA) [13] and reasons preventing the uptake of facility-based HIV testing(Uganda)[14]. Further, two publications did not provide a clear comparison of their observed

numbers of rescheduled/missed visits to pre-pandemic times (USA, Rwanda) [15, 16]. Finally, one publication did not describe the comparator for the reported reductions in HIV tests (-59%), VMMC (-99%), ART initiation (-51%), viral load sample collection (-29%), citing a not accessible rapid assessment of Ministry of Health and Child Care in Zimbabwe [3].

Related to two outcomes: median visits per day and delivered ART doses, one publication[17] did not specify the comparator pre-lockdown period and reported a decrease in median visits per day during March 9 – June 10, 2020 (7 vs 13) and no change in the number of delivered ART doses (thanks to adaptations in care).

From the grey literature search, we count as near misses five conference abstracts not containing enough information on the outcome or periods concerned, so that the reported numbers cannot be put into perspective [18-22]. We neither included rapid assessments of the situation in HIV health care in Europe based on surveys of respondents affiliated with organizations advocating or serving PLHIV, i.e. not directly providing the services nor necessarily using them[23, 24]. Similarly, we did not include a survey mapping the situation from the viewpoint of NGOs and community-based groups serving PLHIV[25].

Viral Hepatitis

During the screening process, 581 and 193 records from 2020 and 2021 searches, respectively, were screened by title and abstract; 49 of them were screened in full text. The main reasons for exclusion were that studies focused on COVID-19 among hepatitis patients or on effects of COVID-19 on liver and associated diseases. Further, treatment guidelines were frequently published. Some economic calculations on future effects of medicinal products were excluded as well as genetic studies and investigations from entirely basic and laboratory science. By full-text screening, one study[26] was excluded due to insufficient reporting. The author mentions 375 patients with chronic active HBV registered at a hospital in Duhok city (Kurdistan region, Iraq) who did not receive the required care for the HBV infection during the lockdown. However, neither the duration of the lockdown nor the missing care is further specified.

Conducting the grey literature search, we found mostly webinars, toolkits for advocacy, patient associations and (patient) information material, position statements and guidance on clinical care for patients with (chronic) hepatitis, liver derangement or transplantations during COVID-19 (e.g. https://easl.eu/wp-content/uploads/2020/04/EASL-ESCMID-COVID-19-Position-Paper.pdf). A link announcing a survey among medical service providers and experts on the impacts of COVID-19 on clinical care or waiting-list management of transplant centres worldwide hinted at future resource of valuable data and evidence. A detailed overview of the found grey literature evidence is in S1 Table B. Except for one, the items found were not included for data extraction due to their limited scientific expression (missing data, personal opinions, web seminars), or because the information included was part of published manuscripts already identified by our systematic search (e.g. the results in [27]). The statements from health care professionals, health policy-makers, and staff of WHO regional offices on experiences or status quo of care during the pandemic reported challenges for maintaining essential hepatitis services, such as effects on specimen transportation, testing and prevention efforts, re-purposed staff and suspension of routine clinic services. Concerns from European countries were raised on missed cases of acute HCV and delayed diagnoses of HCC in hepatitis patients.

Malaria

In the full-text screening, we identified only one publication [28] as a near miss. Its focus is not on evaluating the impacts of COVID-19, so there is no comparison to pre-covid times. The paper reports on digitalized mass ITN distribution in Benin, with the distribution phase being affected by COVID-19. This required quick adjustments, and the campaign ended successfully with 94.16% of the enumerated population receiving ITNs.

S1 Table B Details on grey literature findings regarding viral hepatitis. Note that coinfection with COVID-19 is in general of no interest for our review.

Website	Details on the found materials and reasons for exclusion or indication of inclusion
www.easl.eu	Webinar of a discussion among hepatologists/experts on coping strategies with COVID-19, e.g., challenges for maintaining essential hepatitis services, among them effects on specimen transportation, testing and prevention efforts, re-purposed staff and suspension of routine clinics. Also, concerns from European countries on missed cases of acute HCV and delayed diagnoses of HCC in hepatitis patients are expressed, but no impact measure is stated.<br><br>Link to survey launched in September 2020 and sent to 470 transplant centres to collect and analyse regional practices on how liver transplant patients were affected by the pandemic - no results yet.<br><br>Cross-reference to a survey done by the WHA (see below).

	Toolkit for patient associations, e.g., on how to communicate COVID- information to patients and how to advocate for better treatment of liver disease patients (for media). EASL–ESCMID Position Statement on clinical care for patients, e.g., how healthcare providers can reduce transmission and prioritise patients, links to published research, covered by our systematic search (COVID risk and mortality among patients liver disease/viral hepatitis). – coinfection
www.aasld.org	Several Webinars on COVID-19 and liver disease (management, guidance, treatment, clinical insights and case studies); perspectives on impacts for hepatitis care and personal reports including impact on liver transplantation (US). – personal reports and experiences
www.worldhepatitisalliance.org	Webinars with statements from individuals on COVID-19 and the liver and link to a EASL/WHO discussion on the potential impact of COVID-19 on civil society-delivered hepatitis services and patients. – no evidence on the actual impact<br><br>Member survey on COVID-19 pandemic effects on hepatitis services and patients (results included in the extracted data); accessed 30th September, 2020<br><br>Medical advice for individuals living with hepatitis and being infected with COVID-19. – coinfection
www.HepVoices.org	News/information on community pharmacy that has postponed a HCV service from April 2020 to September 2020, but no impact measure stated: https://hepvoices.org/2020/08/hepatitis-c-service-delayed-by-covid-19-to-launch-on-september-1/, accessed 28th January 2021<br><br>Reference to a summary of statements received from some US health departments on experiences, concerns, requests, and strategies for STI care, incl. viral hepatitis. It was reported that programs have reduced or suspended services and activities in response to COVID-19 due to reduced staff. Also mentioned: suspension of outreach, education, and prevention; clinic closures, reductions in clinical services. https://www.naccho.org/blog/articles/lhd-hiv-sti-and-hepatitis-programs-respond-and-adapt-to-covid-19. Redirected from HepVoices.org, accessed 28th January 2021. – no numerical information<br><br>Reference to a press article on reduction in HBV-birth doses in India “19.4 per cent drop in Hepatitis-B birth doses administered and 31 per cent drop in vaccination sessions held in health facility and outreach sessions from April to June 2020 as compared to same period last year as per Health Management Information System (HMIS), in the light of the COVID-19 pandemic,” https://hepvoices.org/2020/09/19-4-drop-in-hepatitis-b-birth-doses-31-drop-in-vaccination-sessions-in-april-june/, accessed 30th Sept 2020. – included

E. Risk-of-bias assessment tools

We used three different tools for the risk of bias (RoB) assessment according to the type of study. For modelling studies, the tool is based on [29] and we added questions (inspired by [30]) assessing the reporting of the model and sources of data informing the model parameters (S1 Table C). For surveys, we made a minor adjustment to the Risk of Bias Instrument for Cross-Sectional Surveys of Attitudes and Practices [31] based on ideas in [32, 33], see S1 Table D. Finally, for other quantitative studies, we compiled the RoB tool from ideas in Appendices F, G in [34], covering two aspects of external and internal validity each, and including an assessment of possible selective reporting (S1 Table E).

S1 Table C Risk-of-bias tool for modelling studies.

**Modelling studies**	
1.Does the model include real world data?	Yes/No
2. Is the model thoroughly described, or a reference to such a description is given, and are parameter values of the model addressed?	Yes/No
3. Is the model under discussion a dynamic model (transmission model)?	Yes/No
4. Are uncertainty analyses conducted on key assumptions which could have impacted the conclusion?	Yes/No
5. Is the source of the data informing the model based on official (routine) records (hospital, national)? (As opposed to expert opinion or convenience data.)	Yes/No

Overall RoB conclusion	No concerns: all „Yes“<br>Moderate concerns: „No“ only in point 2. and/or 5.<br>Major concerns: all other cases

S1 Table D Risk-of-bias tool for surveys.

**Surveys**	**LR= low risk, MR= medium risk, HR= high risk**
1. Is the source population representative of the population of interest?	LR: random sample from a representative population roster (e.g. national registry or all centres in a country) or taking everyone in the population<br>MR: single centre/city/region or non-random sampling from a registry; random sample from participants in a different trial<br>HR: convenience sample, self-recruitment
2. Is the response rate adequate?	LR: >75%<br>MR: ≤75% and respondents are similar to the target population (or to non-respondents) in important characteristics<br>HR: <40% and no characteristics are reported, or differences between respondents and non-respondents are reported<br>Not reported
3. Is there little missing data within questionnaires?	LR: up to 10%, MR: up to 20%, HR: more than 20%, Not reported
4. Was the questionnaire tested, or alternatively, are the exact questions reported?	LR: pilot testing + some clinimetric properties (e.g. face validity, construct validity, comprehensiveness) assessed in a similar population, or the exact question reported and seeming understandable, simple, addressing what it should<br>MR: only pilot testing, or clinimetric properties in a different population; or the question only rephrased<br>HR: no testing at all, or testing not reported, neither reporting of the exact questions
5. Was the survey administered at a time, when the effect of the exposure could have already manifested?	LR: after 6 or more weeks of the exposure<br>MR: in week 2-6 of the exposure<br>HR: in the first 2 weeks of the exposure

S1 Table E Risk-of-bias tool for quantitative studies.

**Other quantitative studies**	**LR= low risk, MR= medium risk, HR= high risk**
1. Are the population or source area and the data sources well described?	LR: good description of both the population variables and data origin<br>MR: limited description of the population variables and/or data origin<br>HR: insufficient description of the population and the data origin
2. Is the eligible population or area representative?	LR: international, national. MR: regional, multi-centre, HR: local, single centre
3. Are the control intervals and control cohorts appropriate?	LR: comparable cohorts and several previous intervals for comparison (capturing the usual variation in the outcome)<br>MR: comparable cohorts, but only a single interval chosen for comparison and its choice is justified<br>HR: non-comparable cohorts and/or a single comparison interval chosen without comments, or no specification of the comparison
4. Is the observation interval meaningful?	LR: long enough for outcome to occur (clinical) OR data covering lockdown and after-lockdown periods<br>MR: data covering fully lockdown but no after-lockdown period<br>HR: not long enough for outcome to occur (clinical) OR data covering lockdown only partially
5. Is there a chance of a reporting bias?	LR: data presented in its entirety (with respect to the research question of interest)<br>HR: data presented selectively

F. Risk-of-bias assessment results

S1 Fig**. A** shows the risk-of-bias assessment of surveys related to hepatitis, tuberculosis and malaria. Surveys related to HIV are assessed in S1 Fig. B. RoB assessment of other studies concerning hepatitis and tuberculosis is shown in S1 Fig**. C**, and concerning malaria and HIV in S1 Fig**. D**. As to modelling studies, the main reason for concerns was the missing uncertainty analysis (for HIV [35-38], for malaria [35, 37, 39], for tuberculosis [35, 40], for viral hepatitis [41]). Two studies did not used a dynamic model ([37] for HIV, [41] for viral hepatitis). One preprint for HIV [42] did not contain a sufficient description of the model and underlying data sources, but the results seemed to be extended and later published in [43]. Similarly, the description of the data sources behind the model for tuberculosis used in [35] did not clearly state to what extent real world data and official records were utilized.

Risk of bias domains

Hepatitis related surveys	Population	Response rate	Missing data	Questionnaire	Adequate time point
Aghemo, Dig Liver Dis	-	X	!	X	-
Huppe, Z Gastroenterol	+	X	+	X	+
Lemoine, Lancet Gastroenterol Hepatol	-	+	!	-	+
Picchio, Harm Reduct J	-	-	!	-	+
Simoes, Eurosurveillance	-	!	!	+	+
Wingrove, Lancet Gastroenterol Hepatol	-	!	!	-	+

Judgement: + Low - Medium X High ! Unclear

Risk of bias domains

TB related surveys	Population	Response rate	Missing data	Questionnaire	Adequate time point
Khan, medRxiv	X	-	+	+	+
Nikolayevsky, Eur Resp	+	+	X	X	+
Udwadia, Lung India	X	+	X	X	-
Shen, Int J Tuberc Lung Dis	+	!	!	-	+
Cronin, MMWR	+	+	!	X	-
The Global Fund, Mitigating...	-	!	!	X	+

Judgement: + Low - Medium X High ! Unclear

Risk of bias domains

Malaria related surveys	Population	Response rate	Missing data	Questionnaire	Adequate time point
Awucha, Am. J. Trop. Med. Hyg.	X	!	!	+	+
The Global Fund, Mitigating...	-	!	!	X	-
WHO Malaria Report, 2020	X	!	!	X	+

Judgement: + Low - Medium X High ! Unclear

S1 Fig. A Risk-of-bias assessment for surveys related to viral hepatitis, tuberculosis and malaria.

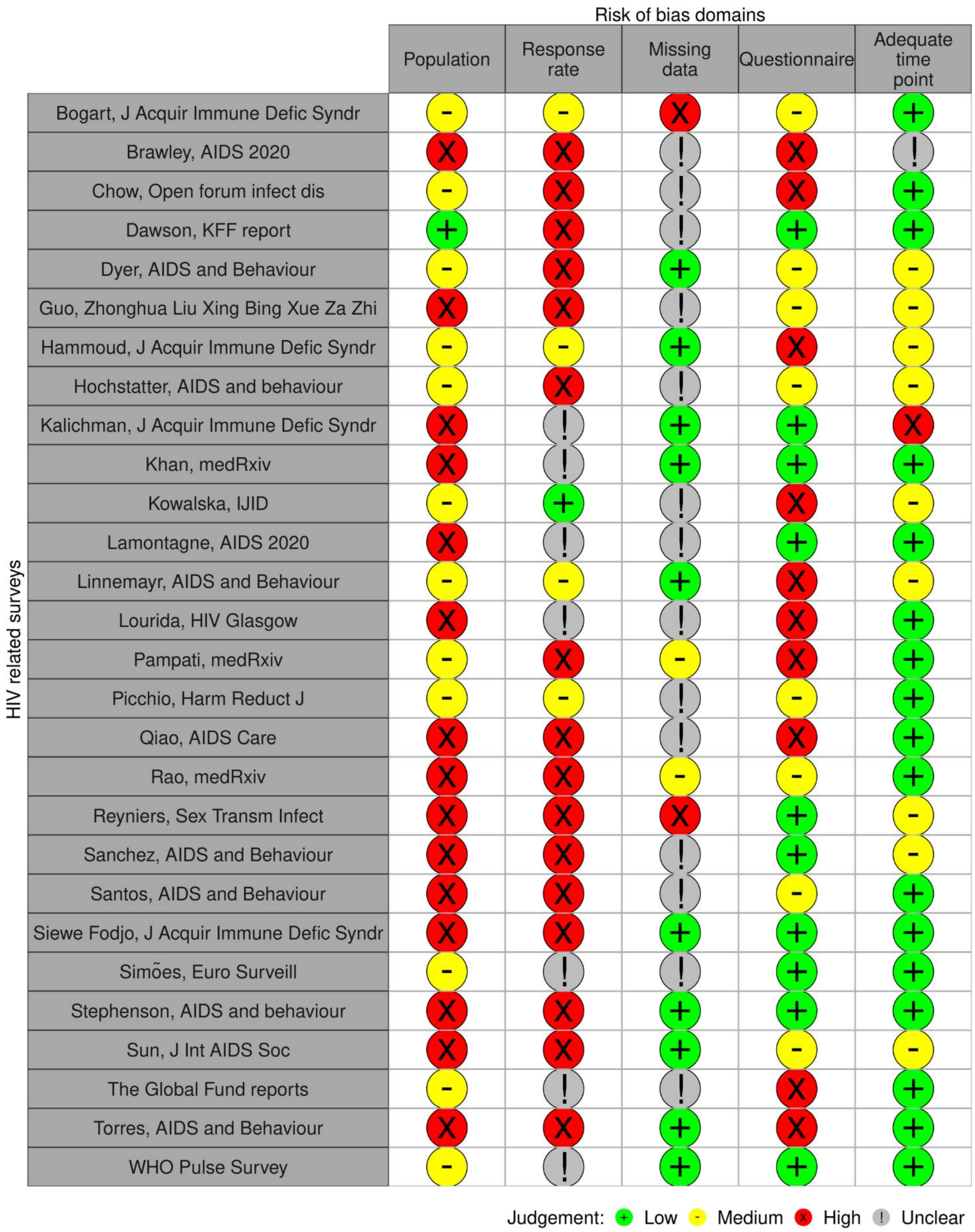

S1 Fig. B Risk-of-bias assessment of surveys related to HIV.

Risk of bias domains

Hepatitis related studies

	Source description	Representativeness	Adequate controls	Observational interval	Reporting bias
Yun, J Med Virol	+	+	+	+	+
Steffen, J of Travel Medicine	-	+	+	+	+
Amaddeo, JHEP Reports	+	-	+	-	+
Tellez, Gastroenterol Hepatol	X	-	X	-	X
McDonald, Euro Surveill	+	+	+	-	+
Bramer, MMWR	+	+	-	-	+
Karimi-Sari, Liver International	X	X	X	-	X
Eshraghian, Lancet Gastronterol Hepatol	+	X	-	-	X
Gaspar, Digestive and Liver Disease	-	X	X	X	X
Toyoda, Hepatology commun	+	-	-	-	+
Toyoda, Gastro Hep	-	X	-	-	X

Judgement: + Low - Medium X High ! Unclear

Risk of bias domains

TB related studies

	Source description	Representativeness	Adequate controls	Observational interval	Reporting bias
Chandir, Vaccine	+	-	-	-	+
Coronel Texeira, JIM	X	+	-	-	X
Datta, TRoySocTropMedHyg	-	X	-	-	+
deSouza, Int J Tuberc Lung Dis	-	-	-	+	+
Fang, Int J Tuberc Lung Dis	-	+	+	+	+
Kadota, Int J Tuberc Lung Dis	-	-	-	X	+
Lebina, SAMJ	-	X	-	+	+
Min, J Kor Med Sci	-	+	-	X	+
Shrinivasan, BMJ Glo H	+	+	-	+	X
Wu, Int J Tuberc Lung Dis	+	-	-	+	+
Behera, Ind J Tuberc	+	+	+	+	+
Bhargava, Ind J Tuberc	+	+	+	-	-
Buonsenso, Pulmonology	-	X	+	-	+
Chen, Eur Resp J	+	+	-	+	-
Chiang, Eur Resp J	+	+	-	-	X
Glaziou, medRxiv	X	-	X	X	X
Iyengar, Ind J Tuberc	-	+	-	X	X
Kwak, Emerg Inf Dis	+	+	+	-	+
Komiya, J Infect	-	+	+	-	-
Lai, J Infect	+	+	+	-	+
Liu, Clin Infect Dis	+	-	+	-	+
Louie, Int J Tuber and Lung Dis	+	X	+	+	+
Magro, Eur Resp J	-	X	-	X	+
Migliori, Emerg Infect Dis	-	+	-	-	+
Union Against TB	-	+	-	+	+
WHO, Global Tuberc Report	+	-	X	+	-

Judgement: + Low - Medium X High ! Unclear

S1 Fig. C Risk-of-bias assessment of quantitative studies related to viral hepatitis and tuberculosis. Note that representativeness is assessed with respect to the global picture and is therefore high if only single centre results are reported.

Risk of bias domains

Malaria related studies	Source description	Representativeness	Adequate controls	Observational interval	Reporting bias
Bell, The Am J Trop Med Hyg	+	+	-	X	+
Buonsenso, medRxiv	+	X	+	-	+
Buonsenso,Frontiers in Pediatrics	+	X	-	-	+
Penjor, Malaria J	X	+	-	+	+
Ranaweera, Malaria J	X	+	-	+	X
Thapa,Research Square	+	+	-	+	+
Guerra, BMJ Global Health	X	X	-	+	X
Feldman,Research Square	X	X	X	+	X
RBM Partnership to End Malaria	X	+	X	+	X
Torres, Am J Trop Med Hyg	-	-	+	+	+
WHO Malaria Report 2020	X	-	+	+	X

Judgement: + Low - Medium X High ! Unclear

Risk of bias domains

HIV related studies	Source description	Representativeness	Adequate controls	Observational interval	Reporting bias
Bogdanic, HIV Glasgow	+	+	+	+	X
Chia, Sex Transm Infect	+	+	+	+	+
Chow, J Acquir Immune Defic Syndr	+	X	+	+	+
Chow, Lancet HIV	-	X	+	+	+
Darcis, Epid anf Infection	+	X	+	+	+
Davey, Lancet HIV	-	X	+	+	+
Ejima, medRxiv	+	+	+	+	+
Giuliani, Sex Transm Infect	X	X	X	+	+
Hickey, AIDS	+	X	+	+	+
Jensen, S Afr Med J	+	+	+	+	+
Junejo, Lancet HIV	-	X	+	+	+
Krakower, AIDS 2020	+	X	-	-	+
Lagat, AIDS and Behaviour	-	-	+	+	+
Maatouk, Sex Transm Infect	X	X	-	+	X
Madhi, S Afr Med J	-	+	+	+	+
Mitsuya, Glob Health Med	+	+	+	+	+
Odinga, Gates Open Res	-	-	+	+	+
Ogirima, AIDS 2020	X	X		+	+
Panagopoulos, HIV Glasgow	-	X	-	+	+
Qiao, AIDS and Behaviour	+	-		X	+
Quiros-Roldan, AIDS Res Ther	+	X	+	+	+
Sánchez-Rubio, Sex Transm Infect	X	-	+	+	X
Shi, Research Square	+	-	+	+	+
Siedner, BMJ Open	+	-	+	+	+
Spinelli, AIDS	-	X	+	+	+
Stanford, AIDS and Behaviour	+	X	X	+	+
Traeger, AIDS	+	+	+	+	+
Tun, AIDS 2020	X	X	-	+	+
UNAIDS reports	X	+	X	+	X
Union Against TB	+	+	-	+	+

Judgement: + Low - Medium X High ! Unclear

S1 Fig. D Risk-of-bias assessment of quantitative studies related to malaria and HIV. Note that the empty grey circles in case of HIV mean "not applicable". Note that representativeness is assessed with respect to the global picture and is therefore high if only single centre results are reported.

G. Tuberculosis

Here we show the detailed findings quantifying the indirect impact of COVID-19 on TB. **S1 Fig. E** shows how the numbers of notifications changed in Americas and Europe. An overview of all the identified studies with respect to the outcome and the type of evidence or of data sources is shown in **S1 Table F**. Further tables detail the impact of COVID-19 on TB notifications(**S1 Table G**), TB-related clinical outcomes(**S1 Table H**), and TB-related service provision(**S1 Table I**), as well as the projected impact on excess cases and deaths from the modelling studies(**S1 Table J**). The entries in the tables are organized according to their geographical location, so a study may appear several times within a table, if it reports on several locations.

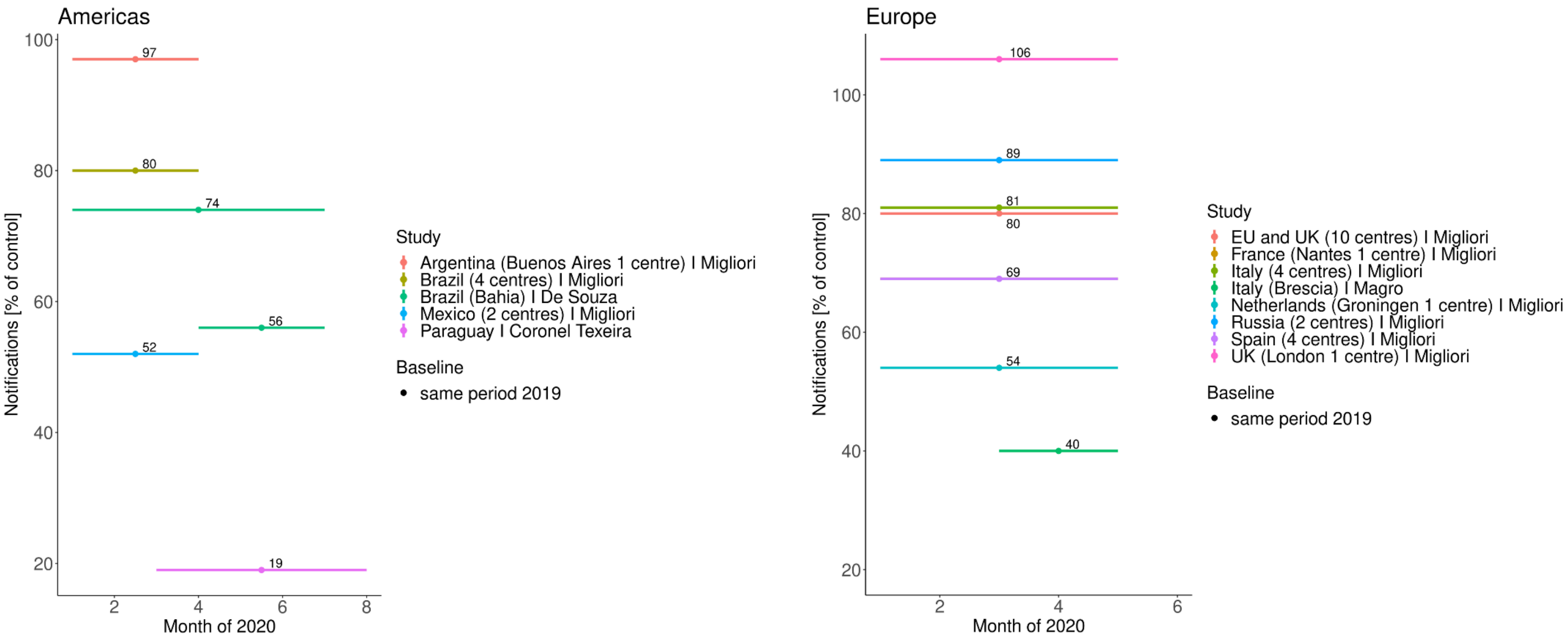

S1 Fig. E TB notifications in Americas and Europe. Overview of notifications registered over a certain period (depicted by the horizontal lines) as percentage of notifications in a control period (referred to as Baseline in the legend). Thus, a value of 20% means a relative decrease of 80%.

S1 Table F Overview of studies related to TB with respect to the outcome and type of evidence/data source.

	National/ regional data	Hospital/program records	Surveys	Grey literature
**Impact on services and service provision**	Louie[44], Chandir[45], Shrinivasan[46], Wu[47]	Migliori[48]	Cronin[49], Khan[50], Nikolayevsky[51], Shen[52], Udwadia[53]	Global Fund[54], WHO[55]
**Notifications/cases**	Behera[56], Bhargava[57], Chen[58], Chiang[59], Coronel Teixera[60], Fang[61], Glaziou[40], Iyengar[62], Kwak[63], Komiya[64], Lai[65], Liu[66], Mohammed[67], de Souza[68], Wu[47]	Buonsenso[69], Datta[70], Magro[71], Migliori[48], Kadota[72], Lebina[73], Min[74]	Shen[52]	Union[75], WHO[55]
**Clinical outcomes**	Liu[66], Mohammed[67], Shrinivasan[46], Wu[47]	Magro[52], Min[74]		Union[75]
**Modeling studies**	Bhargava[57], Cilloni[76], Glaziou[40], Kadota[72], McQuaid[77], Hogan[35]			STOP TB Partnership[78]

S1 Table G Overview of findings describing the impact of COVID-19 on TB notifications. NNV = no numerical value.

Author	Location	Data source	COVID time interval	Covid data	Control interval 1	Control data 1	Control interval 2	Control data 2	Other control intervals	Other control data	Data type	Impact/ summary
**ASIA**	**ASIA**											
Behera[56]	India	national	20/3-3/7/20	362604	20/3-3/7/19	753087	20/3-3/7/18	679222	20/3-3/7/17	56216	Notifications	-51.9%
	India	national	22/3-12/4/20	11367	2-22/3/20	45875					average weekly	-75.2%
Bhargava[57]	India	national	25/3-19/5/20	154258	29/1-24/3/20	380205					Notifications	-59%
Iyengar[62]	India	national	4/20	NNV	4/19	NNV					Notifications	-78%
Datta[70]	Pataudi block, Gurgaon, India	Program data	3/20	46*	3/19	51*					Cases	-9.8%
		Program data	4/20	40*	4/19	62*					Cases	-35.5%
		Program data	5/20	19*	5/19	85*					Cases	-77.6%
Chen[58]	China	National	1/20	67882	1/19	NNV					Notifications	-24%
		National	2/20	44933	2/19	NNV					Notifications	-39%
		National	3/20	73427	3/19	NNV					Notifications	-25%
		National	4/20	85684	4/19	NNV					Notifications	-15%
		National	5/20	83385	5/19	NNV					Notifications	-13%
Shen[52]	China	Hospital (13)	1-3/20	NNV	1-3/19	NNV					Notifications	-25%
	China		4/20	NNV	4/19	NNV					Notifications	-15%

Wu[47]	Shanghai, China	National	24/1-26/3/20	2.4 (2.2-2.6)	1/11/19 - 23/1/20	4.6 (4.3-4.8)					incidence per 1.000.000 person weeks	-47.8%
		National	24/1-26/3/20	2.4 (2.2-2.6)	27/3 - 28/5/20	4.5					incidence per 1.000.000 person weeks	-46.7%
Chiang[59]	China	National	2/20	NNV	2/19	NNV						>-20%
	Malaysia	National	1-5/20	NNV	1-5/19	NNV						-9%
Glaziou[40]	China	Not given	2/20	NNV	2/20	NNV						-20%
	India	National	2-22/3/20	45875	23/3-12/4/20	11367					average weekly	-75%
	Indonesia	Not given	3/20	NNV	1/20	NNV						-68%
Komiya[64]	Japan	National	1/20	950*	1/19	1000*	1/18	1010*	1/17	1100*	Notifications	-5%
			2/20	900*	2/19	1060*	2/18	1065*	2/17	1210*	Notifications	-15.1%
			3/20	980*	3/19	1100*	3/18	1260*	3/17	1265*	Notifications	-10.9%
			4/20	830*	4/19	1150*	4/18	1135*	4/17	1190*	Notifications	-17.8%
Kwak[63]	South Korea	National	weeks 1-8/20	498	weeks 1-8/2015-19	594					average weekly	-16%
		National	weeks 9-18/20	390	weeks 9-18/2015-19	655					average weekly	-40%
Min[74]	South Korea	PPM data	4-6/20	4893	7-9/19	6066					notifications	-19%
Fang[61]	Taiwan	National	1-8/20	5446	1-8/19	5907	1-8/2018	6280	1-8/2017	6623	notifications	-7.8%
Lai[65]	Taiwan	National	weeks 1-20/20	2662	Weeks 1-20/19	3307	weeks 1-20/18	3512	weeks 1-20/17	3563	notifications	-19%
Liu[66]	Jiangsu, China	Regional	1-5/20	6749	1-5/19	10620	1-5/15	14180			notifications	-36%
Migliori[48]	Sidney, Australia	Hospital (1)	1-4/20	52	1-4/19	35					Cases	48.57%
	Singapore	Hospital (1)	1-4/20	16	1-4/19	31					Cases	-48.39%
**AFRICA**												
Buonsenso[69]	Western Area, Sierra Leone	Hospital (1)	4/2020	5	4/19	15	4/2018	13			Cases	-67%
			4/2020	9	4/19	32	4/2018	30			Suspected cases	-72%
Union[75]	Nairobi, Kenya	Hospital (18)	3-8/2020	1334	3-8/19	2041					Cases	-34.6%
	Lilongwe, Malawi	Hospital (8)	3-8/2020	745	3-8/19	962					Cases	-22.6%
	Harare, Zimbabwe	Hospital (10)	3-8/2020	376	3-8/19	504					Cases	-25.4%
	Nairobi, Lilongwe, Harare		3-8/2020	2455	3-8/19	3507					Cases	-30%
	Nairobi, Kenya	Hospital (18)	3-8/2020	8195	3-8/19	17500					Suspected cases	-53.2%
	Lilongwe,	Hospital (8)	3-8/2020	3270	3-8/19	6496					Suspected cases	-49.7%

	Malawi											
	Harare, Zimbabwe	Hospital (10)	3-8/2020	1057	3-8/19	1474					Suspected cases	-28.3%
	Nairobi, Lilongwe, Harare		3-8/2020	12522	3-8/19	25470					Suspected cases	-50.8%
Migliori[48]	Nairobi, Kenya	Hospital (1)	1-4/2020	3733	1-4/19	4270					Cases	-12.6%
	Zinder, Niger	Hospital (1)	1-4/2020	594	1-4/19	704					Cases	-15.6%
	Sierra Leone	Hospital (3)	1-4/2020	114	1-4/19	135					Cases	-16.7%
Mohammed[67]	Dere Dawa, Ethiopia		4-6/2020	110	1-3/20	270	10-12/2019	342	7-9/2019	326	Notifications	-59%
Kadota[72]	Uganda	Hospital (58)	30/3-2/5/20	517 (5 weeks)	13/1-22/3/20	2045 (10 weeks)					Notifications	-43%
			30/3-2/5/20	95-115	13/1-22/3/20	187-236					Notifications	-43%
Lebina[73]	Johannesburg, South Africa	Hospital (1)	27/3-30/9/20	3 (2-4)	1/1/18-26/3/20	14 (5-25)					Average monthly cases	-78.6%
			27/3-30/9/20	10 in 1000	1/1/18-26/3/20	18 in 1000					TB cases per paediatric admission	
**AMERICAS**												
Coronel Texeira[60]	Paraguay	National	03-08/20	NNV	03-08/19	NNV					Notifications	-80.8%
De Souza[68]	Bahia, Brazil		1-7/20	2094	1-7/19	2844					Notifications	-26.4%
			4-7/20	938	4-7/19	1663					Notifications	-43.5%
Migliori[48]	Mexico	Hospital (2)	1-4/20	83	1-4/19	158					Cases	-47.5%
	Buenos Aires, Argentina	Hospital (1)	1-4/20	76	1-4/19	78					Cases	-2.6%
	Brazil	Hospital (4)	1-4/20	268	1-4/19	337					Cases	-20.5%
**EUROPE**												
Magro[71]	Brescia, Italy		1/3-30/4/20	6	1/3-30/4/19	15					Cases	-60%
Migliori[48]	Russia	Hospital (2)	1-4/20	941	1-4/2019	1059					Cases	-11%
	EU and UK [Italy (4), Spain (3), France (1), UK (1), the Netherlands (1)]	Hospital (10)	1-4/20	399	1-4/2019	498					Cases	-20%

	Italy	Hospital (4)	1-4/20	215	1-4/19	266					Cases	-19.2%
	Spain	Hospital (3)	1-4/20	55	1-4/19	80					Cases	-31.2%
	Nantes, France	Hospital (1)	1-4/20	24	1-4/19	35					Cases	-31.4%
	Groningen, Netherlands	Hospital (1)	1-4/20	20	1-4/19	37					Cases	-46%
	London, UK	Hospital (1)	1-4/20	85	1-4/19	80					Cases	+6.3%

*Values are approximated from graph via WebPlotDigitizer (https://apps.automeris.io/wpd/)

S1 Table H Overview of findings regarding indirect impact of COVID-19 on different clinical outcomes related to TB. DOTS= directly-observed therapy schedules, LTFU= loss to follow up.

Author	Location	Data item	COVID time interval	COVID data	Control time intervals	Control data
**ASIA**						
Liu[66]	Jiangsu, China	treatment completion	1-5/2020	53.2%	1-5/2019	61.2%
		treatment success	5/2020	89%	2015-2020	>90%
		MDR screening rate - high risk	5/2020	84%	5/2019	99%
		MDR screening rate - new diagnoses	5/2020	81%	5/2019	98%
Wu[47]	Shanghai, China	Sputum smears	24/1-26/3/2020	86.2%	1/11/2019 - 23/1/2020	95.7%
		Xpert MTB/RIF	24/1-26/3/2020	66.9%	1/11/2019 - 23/1/2020	79.5%
		diagnostic delay	24/1-26/3/2020	18.5 (IQR 9-33)	1/11/2019 - 23/1/2020	15 (IQR 6-29)
Min[74]	South Korea	Sputum smear test coverage	4-6/2020	94.4%	7-9/2019	94.6%
		Sputum culture coverage	4-6/2020	94.1%	7-9/2019	94.2%
		Treatment adherence	4-6/2020	93.3%	7-9/2019	93.4%
		Drug-susceptibility test coverage	4-6/2020	93.6%	7-9/2019	93.6%
		LTFU	4-6/2020	2.1%	7-9/2019	2.6%
		Treatment success	4-6/2020	84.1%	7-9/2019	90.6%
Shrinivasan[46]	India	BCG vaccination	3/2020	NNV	1/2020	NNV
		DOTS patients	4/2020	-50%	2/2020	1
		Treatment success	6/2020	NNV	6/2019	NNV
		TB hospitalisations	6/2020	-66%	6/2019	1
Chandir[45]	Sindh, Pakistan	BCG - fixed location	23/03-09/05/2020	-40.6%	23/09/2019 - 22/03/2020	1
		BCG – outreach	23/03-09/05/2020	-83.5%	23/09/2019 - 22/03/2020	1
		BCG - total	23/03-09/05/2020	-56.6%	23/09/2019 - 22/03/2020	1
**AFRICA**						
Union[75]	Kenya, Nairobi	treatment success	3-8/2020	64%	3-8/2019	67.8%
	Lilongwe, Malawi	treatment success	3-8/2020	96.5%	3-8/2019	96.3%
	Harare, Zimbabwe	treatment success	3-8/2020	63.3%	3-8/2019	80.1%
	Nairobi, Lilongwe, Harare	treatment success	3-8/2020	72.3%	3-8/2019	77.7%
Mohammed[67]	Dere Dawa, Ethiopia	Cure	4-6/2020	11	1-3/20; 10-12/19; 7-9/19	75; 79; 85
		treatment completion	4-6/2020	67	1-3/20; 10-12/19; 7-9/19	92; 147; 194

		LTFU	4-6/2020	5	1-3/20; 10-12/19; 7-9/19	3; 4; 2
		Death	4-6/2020	6	1-3/20; 10-12/19; 7-9/19	10; 5; 10
		Treatment failure	4-6/2020	0	1-3/20; 10-12/19; 7-9/19	0; 2; 0
**EUROPE**						
Magro[71]	Brescia, Italy	LTFU	1/3-30/4/2020	7/65	1/3-30/4/2019	2/76
		Deaths	1/3-30/4/2020	3/65	1/3-30/4/2019	0/76
		Completed treatment	1/3-30/4/2020	10/65	1/3-30/4/2019	11/76
		patients newly entering TB care	1/3-30/4/2020	65	1/3-30/4/2019	76

S1 Table I Overview of published evidence regarding impact of COVID-19 on TB-related service provision. IP= in-patient, OP=out-patient, PPE = personal protective equipment.

**Author**	**Location**	**Data item**	**Time interval (control interval)**	**COVID data (control data)**	**Control interval**	**Control data**
WHO[55]	184 countries	fewer health facilities providing OP care for tb	1-6/2020	32/184, 7/30 high burden		
		fewer health facilities providing OP care for drug-resistant tb	1-6/2020	21/184, 4/30 high burden		
		fewer hospitals providing IP care for tb	1-6/2020	35/184, 9/30 high burden		
		fewer hospitals providing IP care for drug-resistant tb	1-6/2020	33/184, 9/30 high burden		
		reduced number of OP visits for patients with tb	1-6/2020	127/184, 28/30 high burden		
		reallocation of staff	1-6/2020	85/184, 20/30 high burden		
		reallocation of funding	1-6/2020	52/184, 14/30 high burden		
		reallocation of GeneXpert machines	1-6/2020	43/184, 13/30 high burden		
		people with Tb asked to self-isolate at home	1-6/2020	93/184, 14/30 high burden		
Global Fund[54]	104 country agents	Tb service disruption	01/06/2020	78%, 17% high/ very high		
		lab services disruption	01/06/2020	20% high or very high		
		shortage of medical supplies and treatment	01/06/2020	9%		
Khan[50]	64 LMICs	Access for patients to healthcare facility	12/5 - 5/8/2020	40% very hard/impossible		
		Access for provider to healthcare facility	12/5 - 5/8/2020	37% very hard/impossible		
		Access for patients to non-medical support	12/5 - 5/8/2020	31% very hard/impossible		
		TB medical treatment provision	12/5 - 5/8/2020	11% very hard/impossible		
		Diagnostic service provision	12/5 - 5/8/2020	29% very hard/impossible		
Cronin[49]	USA	staffing capacity	4/2020	60-72% partial/ high impact		
		essential services for tb patients	4/2020	52% partial/ high impact		
		essential services for latent tb patients	4/2020	68% partial/ high impact		
		contact tracing	4/2020	64% partial/ high impact		
		testing/treatment of latent tb in populations at risk	4/2020	74% partial/ high impact		
		case reporting and surveillance	4/2020	58% partial/ high impact		
		program evaluation	4/2020	74% partial/ high impact		
		education and training	4/2020	94% partial/ high impact		
		challenges obtaining medication	4/2020	15%	3/2020	7%

		reallocation of resources (e.g. PPE)	4/2020	12%	3/2020	7%
Louie[44]	San Franscisco, USA	B-notification immigrant evaluations	2-5/2020	19	2-5/19;<br>2-5/18	76; 84
Shen[52]	China	change in no. of beds for TB	1-3/2020	-75.7%	1-3/2019	1
		change in no. of beds for TB	4/2020	-43.6%	4/2019	1
		change in no. of staff for TB	1-3/2020	-28.5%	1-3/2019	1
		change in no. of staff for TB	4/2020	-10%	4/2019	1
		hospitals setting strict indications for hospital admission	1-3/2020	38.5%	1-3/2019	0
		hospitals setting strict indications for hospital admission	4/2020	23.1%	4/2019	0
		hospitals setting restrictions for maximum OP visits	1-3/2020	30.8%	1-3/2019	23.1%
		hospitals setting restrictions for maximum OP visits	4/2020	23.1%	4/2019	23.1%
		change in no. of OP visits	1-3/2020	-34%	1-3/2019	1
		change in no. of OP visits	4/2020	-20%	4/2019	1
		change in no. of admissions	1-3/2020	-30%	1-3/2019	1
		change in no. of admissions	4/2020	-18%	4/2019	1
Wu[47]	Shanghai, China	TB clinics closed	24/1-26/3/2020	6/26		
		Tertiary centres halving hospital beds	24/1-26/3/2020	2/4		
Udwadia[53]	Mumbai, India	problems collecting drugs	24/3/2020-	78%		
		problems finding lab for ECG/bloods	24/3/2020-	64%		
		difficulties with transport	24/3/2020-	21%		
		issues with administration of injectables	24/3/2020-	7%		
Nikolayevsky[51]	EU reference laboratories	Impact on services significant/ very significant	11/3-11/4/2020	17/30		
		Impact on services significant/ very significant	11/4-11/5/2020	14/30		
		Impact on services significant/ very significant	11/5-11/6/2020	10/30		
		Impact on training significant/ very significant	11/3-11/4/2020	23/30		
		Impact on research and development significant/ very significant	11/3-11/4/2020	15/30		
		>25% reduction in workload in primary diagnostic services	11/3-11/4/2020	16/27		
		>25% reduction in workload in reference services	11/3-11/4/2020	12/29		

S1 Table J Overview of the modelling studies regarding indirect impact of COVID-19 on TB.

Author	Location	Scenario	Excess cases over 5 years	Excess deaths over 5 years
Cilloni[76]	Worldwide	3m lockdown, 10m restoration; TB transmission -50%	+8% (4.702.300)	+ 12.3% (1044800)
Cilloni[76]/ StopTB[78]	Worldwide	3m lockdown, 10m restoration; TB transmission -10%	+10.7% (6.331.100)	+16% (1367300)
Glaziou[40]	Worldwide	Decrease in case detection by 25% over 3m		+13% (1.660.000)
	Worldwide	Decrease in case detection by 50% over 3m		+26% (3.320.000)
McQuaid[77]	China, India, South Africa	Low reduction in TB transmission, high demand on healthcare		+8-14%

		system		
Hogan[35]	One very high burden setting, one moderate burden setting	Pandemic suppression, high demand on health services		+20%

H. Viral hepatitis

Of 21 records eligible for inclusion and data extraction, 6 were surveys[27, 79-83], 3 modelling studies[41, 84, 85], 11 other studies [86-96] using mostly hospital or notifiable disease records, and one was a press article [97] based on hospital records. The details are presented in **S1 Table K** (modelling studies), **S1 Table L** (surveys) and **S1 Table M** (other studies incl. grey literature sources). Note that the studies are grouped by outcomes and thus some may appear in the table several times, if they report on more than one outcome.

S1 Table K Findings of the modelling studies related to indirect impact of COVID-19 on viral hepatitis. RR= relative risk, HCC= hepatocellular carcinoma, HIC = high-income countries, UMIC = upper-middle-income countries, LMIC = lower-middle-income countries, LIC = low-income countries, UI = uncertainty interval.

Author	Region	Baseline scenario	COVID-19 impacted scenario	Endpoint	Result	Comment
Lai et al. [41]	UK	1-year mortality estimated based on data from 2012-2016 in England, scaled-up to the population of England aged 30+ (35 million individuals)	RR resulting from COVID-19 infection: 1.2, 1.5, 2<br>RR resulting from service change: 1.2, 1.5, 2<br>Infection rate over 1-year: 10%<br>Proportion affected by service change 30%, 70%	Excess deaths for incident liver cancers over a 1-year period	55, 137, 274 at RR 1.2; 1.5; 2, respectively, applied to 10% infected and 30% affected by service change<br>110, 274, 548 at RR 1.2; 1.5; 2, respectively, applied to 10% infected and 70% affected by service change	Mixed direct and indirect effect of COVID-19.<br>Ad-hoc chosen RR representing the service interruption/change.
				Excess deaths for prevalent liver cancers over a 1-year period	26, 65, 131 at RR 1.2; 1.5; 2, respectively, applied to 10% infected and 30% affected by service change<br>52, 131, 262 at RR 1.2; 1.5; 2, respectively, applied to 10% infected and 70% affected by service change	
Abbas et al. [85]	54 African countries	---	6 months of COVID-19, 6-month suspension of vaccination, no catch-up thereafter, unvaccinated children have the same risk of disease as children in a completely unvaccinated population	Benefit/risk ratio of HBV vaccination at 6, 10, 14 weeks<br>(comparing excess COVID-19 deaths resulting from vaccination visits and deaths averted by vaccination up to 5 years of age)	Benefit/risk for household: 1 (95% UI: 0.1 -2)<br>Benefit/risk for vaccinated child: 686 (95% UI: 33 – 4688)	HBV vaccination is done together with vaccination against diphtheria, tetanus, pertussis, Haemophilus influenza type b, Streptococcus pneumonia, rotavirus. Considered together, the benefit/risk ratio for a household is 82 (14 – 261), supporting vaccination without doubt.
Blach et al. [84]	Global (110 countries)	Number of screened patients remains constant on the last known level after 2019, number of patients initiated on treatment annually drops to 50% over 5 years	No patients diagnosed or initiated on treatment in 2020, thereafter reverting to baseline scenario in 2020, resulting in 1-year offset	Excess prevalent infections 2020-2030	623 000 (95% UI: 609 000- 685 000)	No region projected to meet the target for incidence (90% reduction in new infections by 2030), only high-income countries projected to meet the target for mortality (65% reduction in liver-related deaths).<br>Excess liver related death only 50 600 by a 6-month delay scenario, and 25 300 by a 3-month delay scenario.
				Excess incident HCV 2020-2030	121 000 (95% UI: 118 000 – 133 000) distributed between income groups as follows: HIC: 15%, UMIC: 28%, LMIC: 55%, LIC: 2%	
				Excess incident HCC 2020-2030	44 800 (95% UI: 43 800 – 49 300) distributed between income groups as follows: HIC: 45%, UMIC: 23%, LMIC: 30%, LIC: 2%	
				Excess liver related deaths 2020-2030	72 300 (95%UI: 70 600 – 79 400) distributed between income groups as follows: HIC: 41%, UMIC: 21%, LMIC: 35%, LIC: 3%	

S1 Table L Findings of the surveys related to indirect impact of COVID-19 on viral hepatitis. Reported are changes in offer and uptake of hepatitis health services. DAA = direct-acting antiretroviral, LMIC= low-income and middle-income countries, HCC = hepatocellular carcinoma

Author	Country	Population	Outcome	Baseline	COVID-19 period	N	Result	Comment
Hüppe et al[80]	Germany	Centres of the German hepatitis C registry	Liver consultations	----	Mar – May 2020	47	69% reduced or cancelled consultations<br>>50% appointments cancelled by pts	The offer of consultations went back to pre-covid state in July.<br>78% of the centres stated that the pandemic did not worsen the general care for pts with chronic HCV.
			Number of new pts	Mar – May 2019	Mar – May 2020	64	>6 new pts/month on average: 43.8% in 2019 vs 25% in 2020	
			Delayed diagnosis	----	Mar – May 2020	64	HCC: 9.4%<br>Liver decompensation: > 22%	
Simoes et al[79]	Countries of WHO European region	Providers of testing services reached through EuroTEST consortium network	Testing HBV	Mar – May 2019	Mar – May 2020	47	Severe decrease(>50% volume): 63%	Approximate percentages read off from the plots.
					Jun-Aug 2020	48	Severe decrease(>50% volume): 15%<br>Decrease(>10%): 52%, Increase(>10%): 17%	
			Testing HCV	Mar – May 2019	Mar – May 2020	66	Severe decrease(>50% volume): 67%	
					Jun-Aug 2020	70	Severe decrease(>50% volume): 19%<br>Decrease (>10%): 56%, Increase (>10%): 17%	
Aghemo et al[81]	Italy	Members of the Italian Assoc. for the Study of the Liver	Number of beds	Pre-covid	Apr 8 – May 3, 2020 Lockdown	194	Reduction reported by 33%	No quantitative assessment of the reductions, no pre-specified comparison interval.<br><br>26% respondents reported hepatology ward being converted to COVID-19 ward.
			Day hospital				Reduction:47%<br>Complete interruption: 22%	
			Day service				Reduction: 29%<br>Complete interruption: 24%	
			No change in outpatient activity				Chronic hepatitis and compensated cirrhosis: 0.6%<br>HCC: 18%<br>Decompensated cirrhosis: 32%	
			HBV/HCV initiation of antiviral treatment				Postponed: 23%	
			Cirrhosis screening				Reduced: 46%, suspended: 20%	
			Liver transplant				Pre-transplant assessment reduced: 30%<br>Post-transplant reviews postponed: 44%	
			HCC				Reductions in patients starting treatment: 27%<br>No change in treatment activities: 34%	
Wingrove et al[27]	Global, 32 countries	Civil society organisations, other hepatitis service providers	Viral hepatitis testing	---	Mar 30 – May 4, 2020	132 (48% USA)	64% lack of access (reasons: closing of facilities: 46%, fear of Covid-19: 65%)	123 of 131 (94%) reported their services had been effected by the crisis.
			Access to hepatitis medication				Lack of access: LMIC: 52% (15/29) , USA 8% (5/64)	
			Reasons for the lack of access to hepatitis treatment			64	50% (32/64): avoiding facilities due to COVID-19<br>41% (26/64): services redeployed to fight COVID-19	

							Outside USA: 55%(22/40): travel restrictions	
Lemoine et al[83]	Burkina Faso, Tanzania, The Gambia	Retrospectively counted patients in main referral hospitals for chronic hepatitis HBV, HCV (3 from Burkina Faso, 1 from Tanzania, 1 from The Gambia)	Number of new cases	Jan 2020	Apr 2020	5	Burkina Faso: -71% (96 vs 28) , Tanzania: -95% (107 vs 5) , The Gambia: -83% (36 vs 6)	Rapid tests for HBV not disrupted, but shortage of nucleic acid tests for HBV DNA or HCV RNA.<br>Direct-acting antiretrovirals (DAAs) for HCV temporarily unavailable in Burkina Faso.<br><br>New patients and patients in follow-up in 2020 compared against those seen during the same period last year (Jan 1 to April 30, 2019) in The Gambia shown no significant fluctuation
			Number of follow-up patients			5	Burkina Faso: -73% (185 vs 50) , Tanzania: -77% (35 vs 8) , The Gambia: -98% (142 vs 15)	
			Outpatients visits			5	Reduction of more than 70%.<br>In Tanzania and The Gambia, clinics were temporarily closed at the end of March, 2020 in Burkina Faso clinics remained opened the whole time.	
Picchio et al[82]	Spain	Harm reduction service providers	Persons receiving DAA for HCV	Mar – Jun 2019	Mar – Jun 2020	5	Reduction of 82% (88 persons in 2019 vs 16 persons in 2020)	The average number of users decreased by 22% in 2020 (Jan-June) vs 2019 (from 292 to 215).
			HBV, HCV testing			6	Mostly decreases in testing rates per 1000 service users in March, April, May 200 compared to 2019, some centres reported great increase in June (year 2020 compared to 2019).	

S1 Table M Findings of studies relying on hospital or notifiable disease records, registries and databases, when assessing indirect impact of COVID-19 on viral hepatitis.

Author	Country	Data source	Outcome	Baseline	COVID-19 period	Result	Comment
**Vaccination**							
Bramer et al[90]	US Michigan State	Michigan Care Improvement Registry	HBV birth dose	May 2016, 2017, 2018, 2019	May 2020	No change	Less profound declines observed at ages 3, 7, 16, 19, 24 months.<br>Recommended vaccination includes HBV and HAV vaccines.<br>Size of cohorts: 9537 (in 2020), 9269 (average in 2016 – 2019)
			Completed recommended vaccination at 5 months of age	May 2016, 2017, 2018, 2019	May 2020	Declined from 66.6%, 67.4%, 67.3%, 67.9% during 2016-2019, respectively, to fewer than half 49.7% in May 2020	
			Non-influenza vaccine doses administered	Jan – Apr 2018 and 2019 (averaged)	Jan – Apr 2020	Children ≤18 years: decrease by 21.5%, children aged ≤24 months: decrease by 15.5%	
McDonald et al[92]	England	Electronic patient records:	Delivered first doses of hexavalent vaccine (incl.	Week 1 – 17 in 2019	Week 1-17 in 2020	Week 1-17: -3.5% (-3.7% to -3.4%)<br>Week 1-9: -5.8% (-6.0% to -5.5%)	67 116 in 2020, 69 568 in 2019

		The Phoenix Partnership (TPP) SystemOne	HBV vaccine) to infants younger than 6 months			Week 10-12 (transition period): -4.4% (-4.8% to -4.0%)<br>Week 13-15 (physical distancing): -6.7% (-7.1% to -6.2%)<br>Week 16 -17: increase<br><br>(the 95% confidence intervals are calculated using the Wilson method)<br><br>Weeks 13-15: large regional variation, decrease of more than 10% up to increase of 17%.	Modelling suggested a general decrease in vaccination in weeks 1 to 15 in 2020 compared with the same weeks in 2019, which did not accentuate on introduction of physical distancing, but reversed in week 15, with a percent increase in weeks 16 and 17 of 2020 compared to 2019.<br><br>No adjustment for eligible vaccine recipients; birth rates might play a role with less births. Coverage of vaccination is decreasing over previous years in general.
Yun et al[87]	South Korea	Korea Centers for Disease Control and Prevention, Press release April 22, 2020	Vaccination coverage	Jan-Mar 2019	Jan-Mar 2020	Sustained:<br>HBV, 3 doses: 94.6% in 2019 vs 94.1% in 2020<br>HAV, 2 doses: 93.8% in 2019 vs 94.9% in 2020	
Press article	India (unclear if country-wide or state)	Health Management Information System	Hepatitis B birth dose	Apr-May 2019	Apr-May 2020	Reduction of 19.4% in administered doses.<br><br>"19.4 per cent drop in Hepatitis-B birth doses administered and 31 per cent drop in vaccination sessions held in health facility and outreach sessions from April to June 2020 as compared to same period last year as per Health Management Information System (HMIS), in the light of the Covid-19 pandemic." [citation from a website, see the cell to the right]	https://hepvoices.org/2020/09/19-4-drop-in-hepatitis-b-birth-doses-31-drop-in-vaccination-sessions-in-april-june/, accessed 30th Sept 2020
**Incidence of HAV and HBV**							
Yun et al[87]	South Korea	National Notifiable Disease Surveillance System	Incidence of HAV	Jan – Jun 2015 to 2018 (averaged)	Jan – Jun 2020	1736 cases in 2020, corresponds to 14% decline in comparison to pre-years<br>Incidence rate difference significant during intensified social distancing measures (April, May 2020): -0.56 per 100 000 (95% CI: -0.80 - -0.32)	Poisson confidence interval.<br>Nationwide outbreak of HAV in 2019 is a reason for including only years 2015-2018 in the baseline.
			Incidence of HBV	Jan – Jun 2015 to 2019 (averaged)	Jan – Jun 2020	167 cases in 2020, no significant decline	
Steffen et al[88]	Switzerland	Federal Office of Public Health Bulletins	Incidence of HAV	Weeks 18-29 in 2016 to 2019	Weeks 18-29 in 2020	No significant decrease as compared to previous years<br>19 cases in 2020 correspond to 5%	Switzerland in isolation from week 14 to week 24, incubation period of HAV: 4 weeks

						reduction in comparison to mean 2016-2019 (20 cases)	
**Number of patients visits**							
Toyoda et al[94]	Japan (Ogaki)	Electronic medical records	% of kept monthly scheduled visits for HCC surveillance	Jul 2019-Feb 2020	Mar – May 2020	Decline: 279 of 438(63.7%) visits kept in March-May 2020 vs 1236 of 1271 (97.2%) visits kept previously (p<0.0001).<br>Over 95% visits kept for each month up to February 2020, then decline: March: 79.7%, April: 63%, May: 49.1%	One centre, 935 patients with sustained virologic response, who did not have active HCC in July 2019 and continued visiting hospital for HCC surveillance.<br>Decline less pronounced in cirrhosis or advanced fibrosis patients and those with a history of HCC. Before February 2020 the majority of patients who cancelled did not reschedule; from March 2020, most patients did reschedule.
Toyoda et al.[95]	Japan (Ogaki), USA(Stanford), Singapore	Electronic medical records	Chronic HCV visits	Feb 1 – Mar 14, 2018 and 2019<br>Mar 15 – May 1, 2018 and 2019	Feb 1 – Mar 14, 2020<br>Mar 15 – May 1, 2020	Decreasing trend (p=0.022), separate analyses: Stanford: p=0.002, Ogaki: p=0.281	One centre per country, all visits: 14 403, Stanford: 9345 , Ogaki: 3657 , Singapore: 1401<br>Significant decrease in total number of clinic visits from 2018 to 2019 to 2020 (p=0.038; from 5900 in 2018 to 5270 in 2019 to 3233 in 2020) in all three centres together; but: in separate analyses by site significant decrease only in Stanford (p-trend: 0.038) and Singapore (p-trend: 0.026) but not Ogaki.<br><br>p-values from a logistic regression, analysis not well described.
			Chronic HBV visits			Decreasing trend (p=0.003), separate analyses: Stanford: p=0.103, Ogaki: p=0.016, Singapore: p=0.001	
			Visits of patients with HCC and/or cirrhosis			Decreasing trend not significant (p=0.110)	
			Liver imaging			Abdominal ultrasound: decreasing trend (p=0.003), separate analyses: Stanford: p=0.010, Ogaki: p=0.572, Singapore: p=0.753<br>CT/MRI: decreasing trend (p=0.007), separate analyses: Stanford: p=0.021, Ogaki: p=0.838, Singapore: p=0.012	
**Liver related morbidities**							
Amaddeo et al[86]	France (Paris area)	Digital integrated care patient files	Number of patients with HCC presented to multidisciplinary tumor board	Mar 6 – Apr 17, 2019	Mar 6 –Apr 17, 2020	Decrease(221 in 2020 vs 304 in 2019)<br>Decrease over weeks (March 6 –March 26 vs March 27-April 17) in 2020 (p=0.034, Fisher's exact test). Less pronounced decrease for first diagnoses.<br>First diagnosis of HCC: larger tumor size (p=0.002, Mann-Whitney test)	Six academic hospitals in urban area of Paris, patients presented to multidisciplinary tumor board or with programmed surgical or radiological procedure with intention to treat in the studied periods: n=377 in 2019, n=293 in 2020.<br>Early lockdown. For 7% of patients in 2020 the treatment was not realized and so a reliable delay could not be calculated.<br>Most of the patients with a treatment delayed for more than 1
			Rate of treatment in patients with active HCC			Decrease: 56.7% (n =377) in 2019 vs. 43.7% (n = 293) in 2020, with a significant decrease during the second half of the time period in 2020 (p =0.018, Fisher's exact test).	
			Decision-to-treatment interval			No significant difference (2020 vs 2019).<br>Higher rate of patients with a delay longer	

						than 1 month (21.5%, 63 cases in 2020 vs. 9.5%, 36 cases in 2019, respectively; p =0.001, chi-squared test).<br>Recurrent HCC: higher rate of patients with delay above 1 month in 2020 (23.3%, 44 cases vs 4.7%, 11 cases, p<0.001, chi-squared test).<br>Delay or change in treatment strategy was associated only with the period 2020 in multivariate logistic regression (adjusted OR= 9.661 [95% CI: 2.85–32.72]). Similar results for treatment delay above 1 month.	month have a recurrent HCC, suggesting perhaps that physicians tended to delay treatment for patients, whose tumor biology they were familiar with and could adjust the delay accordingly.
Eshraghian et al[89]	Iran (Shiraz)	Patient charts	Hospital admissions for liver-related disorders	Feb 19 – Apr 30, 2019	Feb 19 – Apr 30, 2020	Decrease (230 in 2019 vs 124 in 2020), mean rate per day: 3.23(SD 1.33) in 2019 vs 5.32(SD 3.37) in 2020, IRR 1.85, 95% CI: 1.49-2.30 (Poisson regression)	One hepatobiliary referral centre,<br>IRR = incidence rate ratio<br>MELD = Model for End-Stage Liver Disease<br>Unclear what test some of the p-values are related to.
			Hospital stay duration			Mean duration longer during pandemic (5.32 days (SD 3.37) in 2019 vs 7.44 days (SD 5.42) in 2020, p<0.001)	
			MELD score for liver cirrhosis patients			Higher in 2020 (15.18 (SD 3.45) in 2019 vs 17.07 (SD 4.05) in 2020, p <0.001)	
Gaspar et al[91]	Portugal	Electronic health records and hospital discharge database	Hospital admissions for decompensated cirrhosis	Mar 2 – May 2, 2015 to 2019	Mar 2 – May 2, 2020	No difference (40 in 2020, median 38, range: 34-42 in previous years)	Tertiary academic hepatology centre<br>Unclear what tests the p-values are related to.<br>MELD = Model for End-Stage Liver Disease<br>CPD = Child-Purgh-Turcotte
			Length of hospital stay			No difference (6.5 days (range 1-27) in 2020 vs 8 days (range 1-58) previously, p=0.48)	
			In-hospital mortality			No difference (15% in 2020 vs 11.7% 2015-2019, p=0.57)	
			MELD score			No difference (14 (range 7-32) in 2020 vs 15 (range 7-32) previously)	
			CPD score			No difference (9(range 6-13) in 2020 vs 8 (range 5-14) previously)	
			Readmissions			More in 2020 (47.5% in 2020 vs 27.7% in 2019, p=0.014), associated with shorter index hospital stay.	
Tellez et al[96]	Spain	Organización Nacional de Trasplantes. COVID-19: Impacto en la actividad de donación y trasplantes. 2020.	Liver transplantations	Jan, Feb 2020	Mar, Apr, May 2020	Decrease: 20-30 liver transplants weekly in Jan and Feb 2020 compared to less than 1/3 of that in March and April 2020 and back to 10-15 (half of initial level in 2020) in May 2020.	

Research							
Karimi-Sari et al[93]	Global	PubMed records	Number of records when searching for Hepatitis[tiab]	Jan-Mar 2019	Jan-Mar 2020	Decrease by 995 records (2 808 in 2020 vs 3763 in 2019)	Only a single comparison, no information on the usual variation of the number of records.

I. Malaria

Here we present overview of the results mapping the indirect impact of COVID-19 on malaria as summarized in the main text. **S1 Table N** shows an overview of all included studies with respect to reported outcomes, considered population and geographical area. Further tables compare projected and observed malaria cases (**S1 Table O**), projected and observed malaria deaths (**S1 Table P**) and the modelled impact of interruption of different malaria prevention measures and the observed interruptions (**S1 table Q**).

S1 Table N : **Overview of studies related to malaria with respect to various characteristics.** Note that one study[37] reported both observational and modelled data. Shown are also general trends observed within the studied country/region: increase (↑), decrease (↓), changes in both directions (↕).

	**Modelling studies (n=6+1)**	**Observational studies/reports/surveys (n=12+1)**
**Source**	Systematic search: 5[35, 37, 98-100], Grey literature: 1[101]<br>References: 1[39]	Systematic search: 10 [37, 102-110], Grey literature: 3 [54, 111, 112]<br>References: ---
**Region**	Africa: 6 [35, 37, 98-101] , 118 LIMCs: 1 [39]	Africa: 5 [37, 102, 104, 106, 110], Asia: 4 [105, 107-109], South America: 1 [103], Countries with malaria cases: 3 [54, 111, 112]
**Population**	General population: 6 [35, 37, 98-101], Pregnant women, children<5y: 1 [39]	General population: 11 [37, 54, 103-105, 107-112], Children < 5y: 2 [102, 106]
**Malaria cases**	4: ↑ [37, 98, 100, 101]	10: ↑ [107, 112] , ↕ [37, 106], ↓ [102, 103, 105, 108, 109, 111]
**Malaria deaths**	6: ↑ [35, 37, 39, 98, 100, 101]	3: ↕ [37], ↓ [109], --- [106] (too short observational time)
**Morbidity**	3: ↑ [35, 37, 98]	---
**Impact on malaria prevention measures**	2: [39, 99]	12 [54, 102-112]
**Adjustments of programs**	---	8 [54, 104-106, 108, 109, 111, 112]

S1 Table O Overview of projected and observed impact of COVID-19 on malaria cases. ↑ = increase, ↓= reduction, ↕= both increase and reduction reported in the publication.

Outcome	Modelling studies	Observational studies/reports/surveys	
	**Projected effect**	**Observed effect**	**Provided explanation**
**Malaria cases**	↑ Africa:<br>• Uganda: + 877317 - 6561342[37]<br>• sub-Saharan Africa:<br>⇒ + 365860 - 4.4 million (+ 1 - 14 %) (countries without planned ITN campaigns in 2020) [101]<br>⇒ + 7.7 - 41.9 million (4 - 23 %) (countries with planned ITN campaigns in 2020) [101]<br>⇒ + 5 - 292 million (+ 2 - 113 %) [98]<br>⇒ + 9 - 46.4 million (+ 4 - 22 %) [100]	↑ Asia:<br>⇒ Bhutan: + 7 (+ 17 %): increase in locally transmitted malaria cases from 2 to 20 (+ 36%) [107]	Pandemic-related interruption of prevention measures leads to an increase in locally transmitted malaria cases
		↑ Africa:<br>⇒ Botswana, Burkina Faso, Guinea, Madagascar, Malawi, Namibia, Mozambique, Sao Tome und Príncipe, Zambia, Zanzibar (Tanzania) [112]	No indication of dates or direct causalities
		↕ Africa:<br>• Uganda: + 374107 (+ 45 %, January 2020), + 344031 (+ 52 %, February 2020), - 44194 (- 6 %, March 2020) [37]<br>• Sierra Leone (1 district, children < 5 years): + 5 (+ 10 %) bis + 45 (+ 150 %, compared to January, February, March, May 2018 /2019), - 3 (- 8 %) up to – 27 (- 45 %, compared to April 2018/2019) [106]	Seasonal monthly fluctuations, if applicable
		↓ Africa:<br>• Sierra Leone (1 district, children < 5 years): - 85 (- 40 % clinical diagnosis), - 30 (- 25 % confirmed cases) [102]<br>• Kenia: decrease of 50% [111]<br>↓ Asia:<br>• Cambodia (three provinces, graphical representation): continuous decrease in number of cases since 2019 with increase in testing capacity. In April 2020, abrupt drop in the number of tests carried out, with continuously falling malaria case numbers [105]<br>↓ South America:<br>• Peru (1 region): reduction in 2020 from February to March by 22%, from March to April by 44%, from April to May by 88% and from May to 21 June by 99% [103]	Consequence of the COVID 19 pandemic
		↓ Asia:<br>• Sri Lanka: - 11 (- 32 %) [108]<br>• Myanmar: - 3923 (- 11 %) [109]	Good malaria prevention work
		↓ Africa:<br>• Angola, Benin, Burundi, Central African Republic, Chad, Comoros, Congo, Democratic republic of the Congo, Swaziland, Ghana, Nigeria, South Africa, Tanzania, Zimbabwe [112]	No indication of dates or direct causalities

S1 Table P Overview of projected and observed impact of COVID-19 on malaria deaths. DALY = disability adjusted life-years lost, YLL = years of life lost, ITN = insecticide-treated bednets. ↑ = increase, ↓= reduction, →=no change, ↕= both increase and reduction reported in the publication.

**Outcome**	**Modelling studies**	**Observational studies/reports/surveys**	
	**Projected effect**	**Observed effect**	**Provided explanation**
**Malaria deaths**	↑ Africa:<br>• Uganda: + 3209 to 31046 [37]<br>• sub-Saharan Africa:<br>⇒ + 667 - 50515 (1 - 82 %) (countries without planned ITN campaigns in 2020) [101]<br>⇒ 24573 - 331630 (+ 8 - 102 %) (countries with planned ITN campaigns in 2020) [101]<br>⇒ + 25000 - 696000 (+ 5,9 -165 %) [98]<br>⇒ + 23590 - 382150 (6 - 99 %) [100]<br>⇒ East/ West Africa: + 79 - 2149 per million inhabitants [35]	↓ Asia:<br>• Myanmar: - 3 (-27%) [109]	Good malaria prevention work
		↕ Africa<br>• Uganda: + 379 (+ 123 %, January 2020), + 455 (+ 243 %, February 2020), - 45 (- 19 %, March 2020) [37]	Seasonal monthly fluctuations, if applicable
	↑ LMIC:<br>• + 28 - 152 (+ 1-2 %, maternal deaths, 118 countries) [39]<br>• + 90 - 1170 (+ 0 - 1 %, children under 5 years, 118 countries) [39]	→ Africa:<br>• Sierra Leone (1 district, children < 5 years): 2 deaths until 5/2020 [106]	No statement possible yet, observation period too short
**Malaria associated morbidity (DALY, YLL)**	↑ DALY: Uganda: + 257780 - 2427769 [37]<br><br>↑ YLL:<br>• East/ West Africa: + 4082 - 111097 per million inhabitants [35]<br>• sub-Saharan Africa: + 1 - 34 million [98]	/	/

S1 table Q Projected consequences of interruption of malaria prevention measures on malaria cases and deaths and overview of observed disruptions of the measures.

Outcome	Modelling studies	Observational studies/reports/surveys
**Prevention measures**	• Interruption of mosquito net distribution campaigns causes the largest increase in malaria case numbers and malaria deaths (considered as a single measure) [35, 98].<br><br>• Reduction of access to effective malaria therapy causes the strongest increase in the number of malaria cases and the number of malaria deaths (considered as a single measure) [100, 101].<br><br>• The more prevention measures are reduced or interrupted at the same time, the greater the increase in the number of malaria cases [35, 98, 100, 101].<br><br>• The more prevention measures are simultaneously reduced or interrupted in the modelling, the greater the increase in malaria deaths [35, 39, 98, 100, 101].<br><br>• Modelling COVID-19 pandemic control scenarios: if restrictions are implemented for 12 months, there is the highest increase in malaria deaths if health systems are poorly managed [35, 98].<br><br>• The more the COVID-19 pandemic can be contained, the more mosquito nets can be distributed, the lower the malaria transmission [99].<br><br>• Reduction in health services could lead to a reduction in intermittent malaria prophylaxis in pregnancy [39].	• Interruptions / reduction of prevention measures due to the COVID 19 pandemic or restrictions taken to combat the pandemic.<br>⇒ Logistic problems [54, 103, 104, 107, 110-112]<br>⇒ Supply bottlenecks [110-112]<br>⇒ Reduced headcount due to COVID-19 illness or pandemic deployment [54, 104, 107, 111, 112]<br><br>• Reduced use of health services [102, 103, 105, 110, 111]<br><br>• Reduced supply of or access to health services or hospitals [54, 103, 110-112]<br><br>• No pandemic-related impairment of prevention measures [106, 108, 109]<br><br>• Adaptation of prevention measures<br>⇒ Hygiene measures, provision of protective equipment [54, 104, 105, 109, 111, 112]<br>⇒ Staff training courses [104-106, 109, 111, 112]<br>⇒ Adaptation of the distribution concepts of the campaigns [54, 105, 111, 112]<br>⇒ Information campaigns [105, 109, 111, 112]

J. HIV/AIDS

In this section we present additional figures and findings related to the indirect impact of COVID-19 on HIV prevention and treatment. **S1 Fig. F** details reported reduction in HIV testing volumes and changes in positivity rates, all numerical values used in the plot can be found in **S1 Table U**. **S1 Fig. G** reports on changes in pre-exposure prophylaxis (PrEP) and **S1 Fig. H** in post-exposure prophylaxis (PEP). Further, **S1 Table S** collects the self-reported disruption in HIV services. Finally, to provide detailed numbers to the endpoints discussed in the paper, **S1 Table T** details all the findings related to ART initiations, ART retention and engagement in care and **S1 Table V** collects results of the modelling studies. In addition, assuming 20% loss to follow-up for 6 months and a subsequent return to 1990 mortality levels, with equal mortality across age-groups, and a 55% decline in ART initiations led to 475 319 excess DALYs(disability adjusted life-years) lost for Uganda[37]. For comparison, 405 100 excess DALYs were predicted for 20% incidence of COVID-19 and age-related mortality based on that of China. Please note that the tables are organised according to outcomes, so the same reference may appear several times.

S1 Table R Overview of studies related to HIV with respect to various characteristics. Two studies from Japan[113, 114] evaluated the same data, so we have dropped reporting on [114]. ART=antiretroviral therapy, PrEP = preexposure prophylaxis, PEP = postexposure prophylaxis

	**Health care records (national or regional/ single hospitals or programmes)**	**Surveys**	**Grey literature**
**ART retention and engagement in care**	Europe: 0/2 [17, 115]<br>Americas: 0/2 [116, 117]<br>Africa: 1/0 [118]	Americas: 5 [119-123]<br>Africa: 2 [124, 125]<br>Asia: 2 [126, 127]<br>global: 3 [128-130]	Africa: 1 [131]<br>Asia: 1[132]<br>Europe: 1[133]<br>global: 3 [134-137]
**ART initiations**	Asia: 1/0 [138], Africa: 1/1[37, 118]		global: 1 [135]
**Facility based HIV testing**	Africa: 1/1 [139]/[140]<br>Asia: 2/0 [113, 138]<br>Europe: 0/1[141]<br>Americas: 0/1[142]<br>Australia: 0/1[143]<br>Middle East: 0/ 1[144]	Americas: 3 [122, 145, 146]<br>Europe: 2 [79, 82]	Africa: 1[75]<br>Americas: 3 [147, 148](on PrEP),[149]<br>global: 1 [135]
**New diagnoses**	Asia: 3/0[113, 138, 150], Africa:0/1[37]<br>Europe: 0/2[115, 141]<br>Australia: 0/1[143]		Europe: 3 [133, 151, 152]
**PrEP**	Africa: 0/1 [153]<br>Australia: 0/1[154]	Americas: 4[122, 123, 145, 146]<br>Europe: 1[155]<br>Australia: 2 [156, 157]	Americas: 3[147-149]
**PEP**	Europe: 0/2[158, 159]<br>Australia: 0/1[160]<br>Middle East: 0/1[144]		
**Disruption and uptake of services in general**	Asia: 1/0[113]<br>Africa: 1/0[161]	Asia: 1[162] ,<br>Americas: 1[163]<br>Europe: 1[164], global: 1[50]	Americas: 1[149]<br>Europe: 3[133, 151, 152]<br>global: 1[54, 165]
**Other prevention services**	Africa: 0/2[140, 166]		Africa: 1[167]
**Modelling studies**	Incidence and/or mortality	Africa: 5 [35, 36, 42, 43] culminated in comparison in [168]<br>USA: 3 [38, 169, 170]<br>China: 1 [171]	
	Disability adjusted life years lost	Africa: 1 [37]	

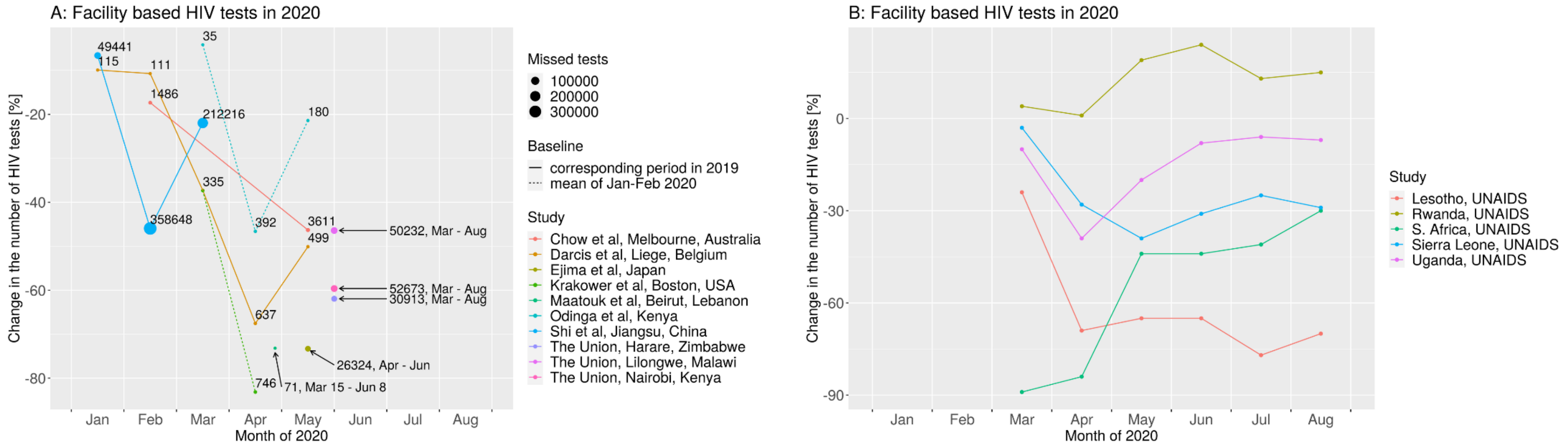

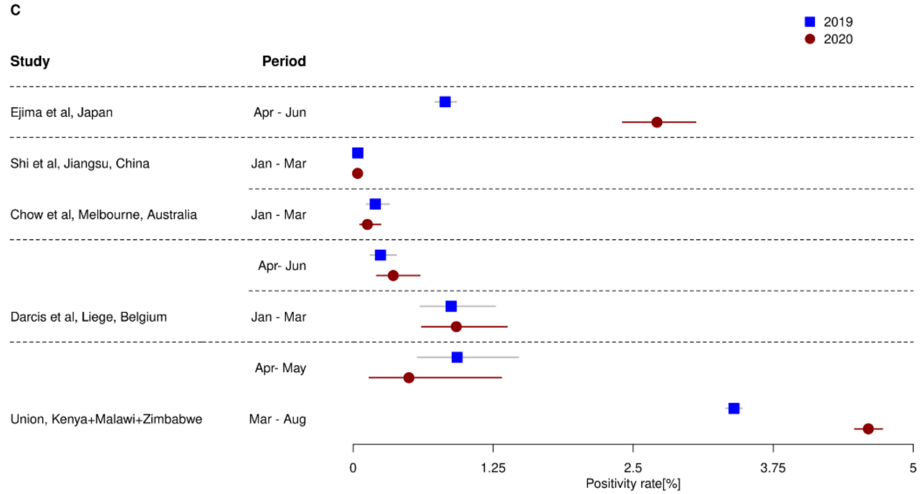

A. Changes in facility-based HIV testing volumes. Shown are the % and absolute decrease in the number of HIV tests. Note that using the mean of Jan-Feb 2020 as the baseline does not account for possible seasonality. The WHO declared COVID-19 to be a pandemic on March 11, 2020, whereas in China the lockdown measures were introduced already in late January 2020. The 85% reduction reported by Krakower et al in April 2020 relates to HIV testing on PrEP and can be compared to 59% of 189 PrEP prescribers in the USA reporting skipping HIV testing on PrEP refill [148].

B. Percent change in HIV testing volumes as read off the plots reported by UNAIDS[135]. In this case the comparison is made to the average of Jan-Feb 2020, so the results may be confounded by seasonality. For example, if we calculate the same percent change for March–August 2019 relative to the average of Jan-Feb 2019 from data reported for one centre in Liege, Belgium[141], we would obtain a decrease between 7.5 to 20% for the whole period.

C. Positivity rate as observed by the different studies in 2020 and 2019. Shown are the point estimates and 95% confidence intervals based on the Agresti-Coull method for binomial proportions.

S1 Fig. F Reported reduction in HIV testing volumes and changes in the positivity rate. The underlying numbers can be found in **S1 Table U**.

PrEP

S1 Fig**. G** shows the self-reported decrease in usage of PrEP and difficulties accessing PrEP. The main reasons for stopping PrEP in Brazil were impediments with refill (47%), followed by sex abstinence (40%). In all other studies the main reason for stopping or reducing PrEP seemed to be a lower perceived HIV risk/decrease in sexual activities with casual partners. Problems accessing PrEP as a reason for stopping were reported only by 7%[156] , 8% [148], 11% [157]. A single centre in Boston [147] reported a decline in PrEP patients, PrEP initiations and a large increase in PrEP refill lapses compared to the mean of January-February 2020(3188 patients, 107 initiations, 141.5 lapses). Namely, in March 2020, there were 2% fewer patients, 44% fewer initiations and 44% more lapses, whereas in April 2020, there were 6% fewer patients, 68% fewer initiations and 187% more lapses. From Australia, a 24% decrease in the mean weekly number of prescriptions (Apr-June 2020 vs Jan 2019-March 2020) was reported[154] and a specific programme in South Africa observed a 68% increase in the rate of missed PrEP visits of pregnant women (from 34% of missed visits pre-lockdown to 57% of missed visits during lockdown) [153].

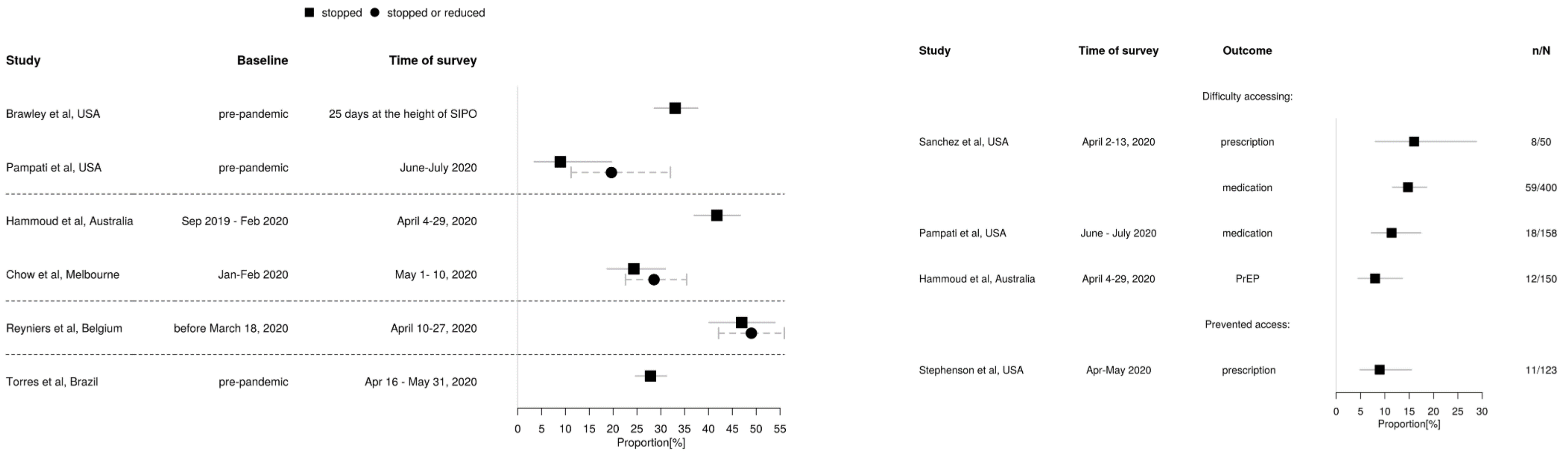

S1 Fig. G Impact on PrEP usage. Left: Proportions of respondents reporting changes in PrEP use. Shown are also the 95% confidence intervals based on the Agresti-Coull method. Right: Proportions of respondents reporting difficulties accessing PrEP. Shown are also the 95% confidence intervals based on the Agresti-Coull method. For comparison, 25% of 169 Ryan White grantees (USA) reported a decreased ability to provide PrEP, and 47% reported a decreased demand for it during the pandemic (until August 2020) [149], whereas in another survey[148] 95% of 189 U.S. prescribers declared the ability to prescribe PrEP also during the shelter-in-place order. For completeness, here are the absolute numbers underlying the left plot: Brawley et al: 135/409, Pampati et al: 5/56 and 11/56, Hammoud et al: 167/400, Chow et al: 46/189 and 54/189, Reyniers et al: 93/198 and 97/198, Torres et al: 204/733.

PEP

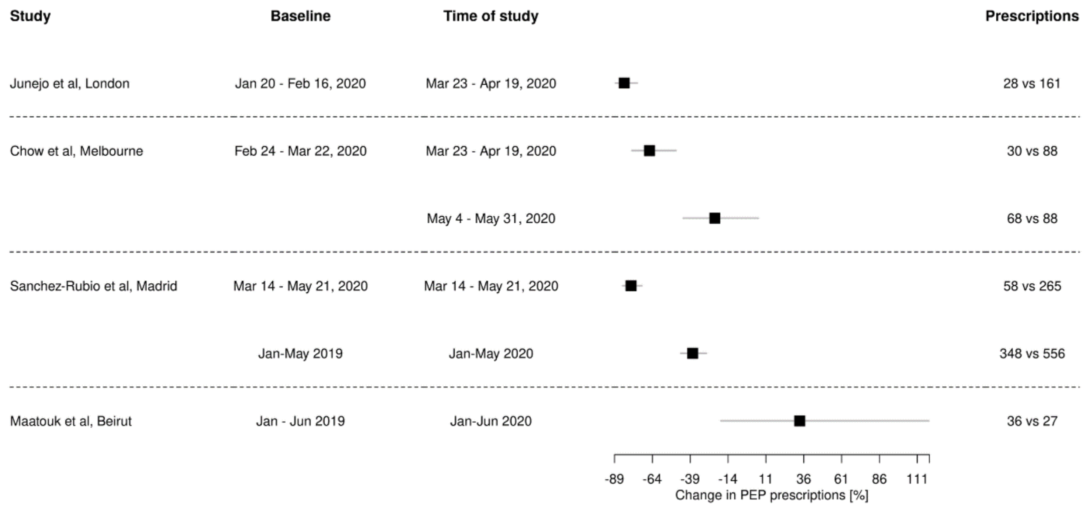

S1 Fig. H Reduction in PEP prescriptions. Shown are 95% confidence intervals based on the assumption of a Poisson distribution for the observed counts. Although the numbers were not reported, Sanchez-Rubio et al. [159] commented that the numbers started to increase even before the lockdown was at the end, which is what Chow et al. [160] observed as well.

Other prevention services

Compared to the mean outreach contacts of 5016 in 3 centres in Kenya [140] in Jan-Feb 2020, the programme registered a decrease of 2%(March 2020), 30%(April 2020) and 13%(May 2020) in the outreach contacts. Similarly, an assisted partner notification programme in the same country observed a decrease of 8%(March 2020) and of 36%(Apr 2020) in female index enrolments as compared to the monthly mean of 160 enrolments in Jan-Feb 2020, across two centres involved[166]. Regarding VMMC, countries reported decreased numbers of circumcisions in April, May, June[167] than in January 2020(most notably Lesotho, South Africa, Mozambique, Botswana and Zimbabwe). Without any quantification, another report of UNAIDS[135] mentions that by late 2020, the VMMC programmes in Kenya, Rwanda, Botswana and South Africa were returning to normal.

Disruption and uptake of HIV services in general

S1 Table S shows the self-reported service interruptions. In addition, a decrease of overall outpatients visits was noted by several authors: a decrease of 64% in the second quarter of 2020 in Japan[113], of around 60% in both March-April 2020 and May-June 2020 in one centre in Greece[152], always compared to the corresponding period in 2019. In Croatia, the weekly mean went from 46(February 2020) to 22.5(March 2020)[133]. In contrast, an immediate increase in the mean visits/clinic /day of 8.0 (95%CI: 2.3-13.7) following the introduction of level 5 lockdown was observed in KwaZulu Natal[161]. This increase was followed by a slight steady decrease until the start of level 4 lockdown, when another stepwise increase of 11 visits/clinic/day (95% CI: 4.2 – 17.8) was observed. Without being specific about the qualitative assessment, as of June 1, 2020, The Global Fund reported 18% of 106 countries experiencing high to very high disruption to HIV services, and 66% experiencing moderate disruptions[54]. The situation seemed to improve only very slightly, with still 55% experiencing moderate disruptions in December [165].

S1 Table S Self-reported HIV service interruptions.

**Country**	**Disruption**	**Country**	
Guangxi, China	Of 1029 professionals [162]:<br>• 59.5% report shortage in personnel<br>• 48.7% report conflict between Covid and HIV tasks<br>• 25% report their clinic has no capacity to maintain HIV services	Europe	Of 19 countries represented by single individuals in March 2020 [164]:<br>• 16%: shorter hours or reduced staff<br>• 53%: only ART distribution(no visits, no blood tests)<br>• 58%: physicians sharing Covid-19 duties<br>Of 9 HIV clinics in Greece[151] asked in June 2020:<br>• 25% cancelled scheduled visits during lockdown completely<br>• 50% covered only emergencies and prescriptions during lockdown
Ryan White grantees, USA	Of 27 clinics in South Carolina in March 2020[163]:<br>• 26% : complete interruption<br>• 56% partial interruption<br>Of 169 grantees in the USA covering the period up to Sep 2020[149]:<br>• 28%: shutting down most or all HIV prevention services<br>• 5%: shutting down most or all HIV treatment services	Global	Of 346 professionals[50] interviewed in May-August 2020:<br>• 16%: experienced serious* challenges in provision of standard treatment/medicine<br>• 33% experienced light** challenges in provision of standard treatment/medicine<br>• 24%: experienced serious* challenges in provision of routine diagnostic services<br>• 36%: experienced light* challenges in provision of routine diagnostic services<br>* it is much harder, it is very difficult or impossible<br>**it is slightly harder

S1 Table T Overview of studies reporting on ART retention and engagement in care. PLWH = people living with HIV, SUD = substance use disorder, MSM = men who have sex with men, AMIS = American Men's Internet Survey, RCT = randomized controlled trial, TGNB = transgender, non-binary individuals

**Detailed outcome**	**Country**	**Population**	**Period**	**Baseline**	**Result**	**Author**
**ART adherence**						
Electronically measured adherence	USA (Los Angeles, California)	Black population living with HIV (from a parent RCT studying motivational interviewing intervention to improve ART adherence)	May -July 2020	Previous 1-month measurement closest to the COVID-19 survey for each participant	OR 0.59 for good adherence (i.e. >80% doses taken per 1 additional experienced negative impact of the pandemic)	Bogart et al[119]
3-item self-reported instrument for retrospective adherence converted into scale 0-100	USA (Atlanta, Georgia)	PLWH 20-37 years old screened positive for substance use (participants of ART adherence study)	Month after onset of COVID-19 protective actions (median 9 months in the study)	Previous month	Significant improvement in the mean adherence score in the month after onset of COVID-19 protective actions (from 52.8 to 77.6)	Kalichman et al[120]
Missed ART dose on 2 or more days of the week	USA (Wisconsin)	PLWH, SUD at high risk of treatment failure	Mar 24 – May 4, 2020	Jan 31 – Mar 12, 2020	OR 2.81 during pandemic, p=0.084	Hochstatter et al[121]

Difficulties with ART refill (possibly including inability to refill)	USA	MSM (AMIS participants)	Apr 2 – 13, 2020	---	8/121 =7%	Sanchez et al[122]
	Global (Belgium 32.2%, Brazil 28.1%, Eastern Europe 18.6%)	PLWH (online questionnaire)	Apr 9 – May 17, 2020	---	29/317=9%	Siewe Fodjo et al[129]
	Global(mostly Brazil, France, Mexico, Taiwan, Russia)	Cisgender gay men and other MSM (networking app Hornet)	Apr 16 – May 4, 2020	---	84/455=18%	Santos et al[128]
	Brazil	MSM and TGNB (Hornet, What's App, facebook)	Apr 16 – May 31, 2020	---	138/800=17%	Torres et al[123]
Limited access to ART	138 countries (Russia, Turkey, France, Brazil,…)	LGBT+(online)	Apr 15 – May 18, 2020	---	21% (from around 2200-2300)	Lamontagne et al[137]
Unable to get ART refill	Western Kenya	Adolescent (10-24 years) living with HIV (ongoing cohort study to improve outcomes)	10 first weeks of COVID-19	---	15/486=3%	Dyer et al[124]
	Global(mostly Brazil, France, Mexico, Taiwan, Russia)	Cisgender gay men and other MSM (networking app Hornet)	Apr 16 – May 4, 2020	---	20/455=4%	Santos et al[128]
	USA (Los Angeles, California)	Black population living with HIV (from a parent RCT studying motivational interviewing intervention to improve ART adherence)	May – July 2020	---	6/101=6%	Bogart et al[119]
No access to ART	138 countries (Russia, Turkey, France, Brazil,…)	LGBT+(online)	Apr 15 – May 18, 2020	---	7% (from around 2200-2300)	Lamontagne et al[137]
Decreased ability to adhere to ART	USA	MSM (AMIS participants)	Apr 2 – 13, 2020	Pre-covid	6/121=5%	Sanchez et al[122]
	Uganda	HIV positive adults (RCT participants in Mildmay Uganda clinic)	Apr 6 – 17, 2020(personal communication)	Pre-covid	14/100=14%	Linnemayr et al[125]
	Global (Belgium 32.2%, Brazil 28.1%, Eastern Europe 18.6%)	PLWH (online questionnaire)	Apr 9- May 17, 2020	Pre-covid	11/312=4%	Siewe Fodjo et al[129]
Risk of ART interruption = having not enough medication under current traffic and movement restrictions	China	PLWH on ART (online questionnaire)	Feb 5-10, 2020	----	331/1014=33%	Guo et al[127]
Risk of ART interruption	China	PLWH (recruited through HIV-	Feb 5-17, 2020	----	917/5084=18%	Sun et al[126]

= medication for less than 10 days without a clear way for refill		themed WeChat public accounts)				
Disruptions in the number of people on ART	Global	PLWH on ART (country programme data reported to HIV Services Tracking Tool)	Mar – Sep 2020	Jan – Feb 2020	16/25 countries no disruptions, some have seen drops in the number of people on ART:<br>Peru, -14%, July 2020<br>South Africa: -5/-6% in July/August 2020,<br>Dominican Republic: up to -10% Apr – Sep 2020	UNAIDS[135]
**Viral load suppression**						
Unsuppressed viral load	USA (San Francisco)	PLWH attending a safety net clinic (Ward 86)	Apr 1 – Apr 30, 2020	Dec 1, 2019 – Feb 29, 2020	OR 1.31 (1.08– 1.53), n=1766	Spinelli et al[116]
	USA (San Francisco)	Homeless PLWH in a low-barrier HIV primary care programme (Ward 86)	Mar 17 – Aug 16, 2020	Oct 17, 2020 – Mar 16, 2020	OR 1.19 (0.78-1.82), n=66	Hickey et al[117]
Number of avireamic patients	Italy (Rome)	HIV patients (HIV/AIDS unit of S. Gallicano Dermatological Institute)	Mar 9 – Aug 20, 2020	Pre-lockdown	95.8% of 506 aviraemic before lockdown remained avireamic	Giuliani et al[17]
**ART dispensation**						
Pick-up coverage	Lagos, Nigeria	Clients of Centre for Integrated Health Programme	Apr – May 2020	---	97% of all eligible for pick-up received ART refill at the end of the lockdown period thanks to adaptation of services	Ogirima et al[131]
Number of ART dispensations	Italy (Brescia)	HIV patients (tertiary referral center for Infectious Diseases and HIV in Brescia)	Mar-Apr 2020	Oct – Nov 2019	-23.1% (-33.6% in March, -12.9% in April)	Quiros-Roldan et al[115]
Weekly number of individuals receiving treatment	Nepal	Patients in sites supported by LINKAGE project and other partners	Mar 22 – May 16, 2020	Jan 26 – Mar 21, 2020	Ca 800-1200 vs ca 1700-2000(baseline)	UNAIDS[134]
**ART consultations**						
Number of ART consultations	Myanmar	ART patients in a primary care clinic	Apr 2020	Apr 2019	6630 vs 6009 (baseline)	Tun et al[132]
**Viral load tests**						
Number of viral load tests	Croatia	PLWH treated in Croatia	Mar- Jun 2020	Mar –Jun 2019/2018	ca -30% (545 vs 777/762)	Bogdanic et al[133]

	South Africa	Tests carried out by the sole laboratory service provider to public sector	Mar 27 – Apr 30, 2020/May 1 – June 1, 2020	Feb 1 – Mar 26, 2020	-22%/-5% in weekly averages	Madhi et al[118]
**ART initiations**						
Number of ART initiations	China(Jiangsu)	PLWH starting ART (data from Comprehensive response Information Management System for HIV/AIDS in China)	1st quarter 2020	1st quarter 2019	-34% (208 vs 315)	Shi et al[138]
More sustained disruptions in ART numbers	Global	PLWH starting ART (country programme data reported to HIV Services Tracking Tool)	Mar – Aug 2020	Jan-Feb 2020	21/28 countries, individual monthly changes in the range: -45%(Sierra Leone) - +88% (Nigeria)	UNAIDS[135]
Number of ART initiations(weekly mean)	Uganda	PLWH starting ART (recorded by PEPFAR, Uganda ministry of Health and partners)	Apr 2020 (weeks 14-16)	Oct 2019 – Mar 2020	-60% (1096 vs 2721) [reported also -37% in week 23, June 2020]	Bell et al[37]
CD4+ tests (proxy for initiation)	South Africa	Tests carried out by the sole laboratory service provider to public sector	Mar 27 – Apr 30, 2020/May 1 – June 1, 2020	Feb 1 – Mar 26, 2020	-33%/ -15% in weekly averages	Madhi et al[118]
**Keeping HIV appointments**						
Rate of missed visits	USA (San Francisco)	PLWH attending a safety net clinic (Ward 86)	Apr 1 – 30, 2020	Dec 1, 2019 – Feb 29, 2020	599/1997(30%) vs 1287/4153(31%, baseline), OR 0.91 (0.77-1.09)	Spinelli et al[116]
	Italy (Brescia)	HIV patients (tertiary referral center for Infectious Diseases and HIV in Brescia)	Mar-Apr 2020	Oct – Nov 2019	94/1162(8%) vs 60/1217(5%, baseline)	Quiros-Roldan et al[115]
Keeping regular visits (visit each month)	USA (San Francisco)	Homeless PLWH in a low-barrier HIV primary care programme (Ward 86)	Mar 17 – Aug 16, 2020	Oct 17, 2020 – Mar 16, 2020	OR 0.66 (0.39-1.11)	Hickey et al[117]
Confidence to keep the next HIV appointment (scale 1-7)	USA (Wisconsin)	PLWH, SUD at high risk of treatment failure	Jan 31 – Mar 12, 2020	Mar 24 – May 4, 2020	Decrease in mean score from 6.89 to 6.5, p=0.021(Poisson regression)	Hochstatter et al[121]
**ART services interruptions**						
Disruption to services	Global (all Regions, but the Region of the Americas)	Data collected by WHO regional offices through facilitated discussion with key informants within the national ministry of health	May-Jul 2020	Pre-covid, unspecified	partial disruption(5-50% pts not treated as usual): 31% of 101<br>sever disruption(>50% of pts not treated as usual): 1% of 101	WHO[136]

**Difficulties accessing HIV provider**						
Unable to access provider	Western Kenya	Adolescent (10-24 years) living with HIV (ongoing cohort study to improve outcomes)	First 10 weeks of COVID-19	---	85/486 =17%	Dyer et al[124]
	Global(mostly Brazil, France, Mexico, Taiwan, Russia)	Cisgender gay men and other MSM (networking app Hornet)	Apr 16 – May 4, 2020	---	111/473 =23%	Santos et al[128]
	Global (about one third from Russian Federation)	PLWH (users of Hornet, gay social networking app)	Apr 16 – May 24, 2020	---	218/1105=20%	Rao et al[130]
Trouble making/keeping appointment	USA	MSM (AMIS participants)	Apr 2 – 13, 2020	---	24/121=20%	Sanchez et al[122]
COVID-19 impacts my ability to come to clinic	Uganda	HIV positive adults (RCT participants in Mildmay Uganda clinic)	Apr 6 – 17, 2020	---	76/100=76%	Linnemayr et al[125]

S1 Table U Overview of findings related to HIV testing and new diagnoses. MSM=men who have sex with men

**Detailed outcome**	**Country**	**Population**	**Period**	**Baseline**	**Result**	**Author**
**Facility based HIV tests**						
Change in the number of tests	Australia (Melbourne)	Data from Melbourne Sexual Health Centre	1st quarter | 2nd quarter of 2020	1st quarter | 2nd quarter of 2019	-17% (7083 vs 8569) | -46% (4187 vs 7798) [comparison between the respective quarter of 2020 and 2019]	Chow et al[143]
	Belgium (Liege)	Data from Liege University Hospital	Jan | Feb | Mar | Apr| May 2020	Jan | Feb | Mar | Apr| May 2019	-10% (1046 vs 1161) | -11%(925 vs 1036) | -37% (634 vs 1011) |-68% (306 vs 943)| -50% (497 vs 996)<br><br>[comparison between the respective month in 2020 and 2019]	Darcis et al[141]
	Lebanon (Beirut)	Patients at Clemenceau Medical Center	Mar 15 - Jun 8, 2020	Mar 15 - Jun 8, 2019	-73% (26 vs 97)	Maatouk et al[144]
	Kenya (Kisumu, Kiambu, Momabasa)	combined data from 3 prevention programmes aimed at MSM	Mar | Apr | May 2020	Jan-Feb 2020	-4% (806 vs 841) | -47%(449 vs 841) | -21% (661 vs 841) [comparison between the respective month in 2020 and monthly mean of Jan-Feb	Odinga et al[140]

					2020]	
	Kenya	Data from 18 health facilities in Nairobi	Mar-Aug 2020	Mar-Aug 2019	-60% (35676 vs 88349)	Union[75]
		Data from 8 health facilities in Lilongwe	Mar-Aug 2020	Mar-Aug 2019	-46% (57972 vs 108204)	Union[75]
	Zimbabwe	Data from 10 health facilities in Harare	Mar-Aug 2020	Mar-Aug 2019	-62% (18987 vs 49900)	Union[75]
	China(Jiangsu)	Screening tests, data from Comprehensive response Information Management System for HIV/AIDS in China	Jan | Feb | Mar 2020	Jan | Feb | Mar 2019	-7% (699310 vs 748751) | -46% (422040 vs 780688)| -22% (754335 vs 966551) [comparison between the respective month in 2020 and 2019]	Shi et al[138]
	Japan	Data from quarterly reports from the National AIDS surveillance Committee	2nd quarter of 2020	2nd quarter of 2019	-73% (9584 vs 35908)	Ejima et al[113]
	Lesotho	country programme data reported to HIV Services Tracking Tool	Mar | Apr |May| Jun| Jul | Aug 2020	Jan-Feb 2020	-24% | -69% | -65% | -65% | -77% | -70% [comparison between the respective month in 2020 and monthly mean of Jan-Feb 2020]	UNAIDS [135] (approximately read off the plots)
	Sierra Leone				-3% | -28%| -39%| -31%| -25%| -29% [comparison between the respective month in 2020 and monthly mean of Jan-Feb 2020]	
	South Africa				-89%|-84%|-44%|-44%|-41%|-30% [comparison between the respective month in 2020 and monthly mean of Jan-Feb 2020]	
	Uganda				-10%|-39%|-20%|-8%|-6%|-7% [comparison between the respective month in 2020 and monthly mean of Jan-Feb 2020]	
	Rwanda				4%|1%|19%|24%|13%|15%	

					[comparison between the respective month in 2020 and monthly mean of Jan-Feb 2020]	
Change in the number of HIV tests on PrEP	USA(Boston, MA)	Data from Ben Israel Deaconess Medical Center	Mar |Apr 2020	Jan – Feb 2020	-37% (562 vs 897) | -83% (151 vs 897) [comparison of the respective month in 2020 with monthly mean of Jan-Feb 2020]	Krakower et al[147]
Uptake of HIV testing at birth	South Africa (KwaZulu-Natal)	Data from District Health Information System	Mar | Apr | May | Jun 2020	Jan –Feb 2020	Uptake of 89.6%| 88.2%| 80.1%|91.1%, baseline: monthly mean 93.9% 95% CI: 92.4 – 95.4	Jensen et al[139]
HIV testing rate in emergency department	USA (Chicago,Illinois)	Data from Emergency Dept., University of Chicago	Mar 5 – Apr 18, 2020	Jan 1 – Mar 4, 2020	1789/8616(21%) vs 3247/14524(22%, baseline), no significant difference	Stanford et al[142]
Being prevented from HIV testing	USA	Gay, bisexual and other MSM(recruited through facebook, Instagram, Grindr app)	Apr – May 2020	Pre-pandemic	32% of 518	Stephenson et al [145]
Experienced difficulties getting tested when tried	USA	MSM (AMIS participants)	Apr 2 – 13, 2020	---	18% of 286	Sanchez et al[122]
	USA	MSM on PrEP(recruited online for a cohort study examining trajectories of PrEP use, Oct 2019 – Jul 2020)	Jun-Jul 2020	---	24% of 45	Pampati et al[146]
Decreased demand for testing (since the beginning of pandemic)	USA	Ryan White HIV/AIDS Program medical provider grantees	Aug 2020	---	64% of 169	KFF[149]
Decreased ability to test (since the beginning of pandemic)					61% of 169	
Skipping HIV testing on PrEP refill	USA	PrEP providers from American Academyof HIV Medicine's database	25-day period of shelter-in-place orders implementation	---	59% of 189 providers	Brawley et al[148]
Severe reduction(>50%)	WHO European region	Laboratories, primary and	Mar-May 2020	Mar - May 2019	60% of 86	Simoes et al[79]

in HIV testing volumes		secondary level care clinics, community sites, national level public health institutions, ministries of health (contacted through EuroTEST consortium network)	Jun-Aug 2020	Mar – May 2019	20% of 86	
HIV testing rates per 1000 service users	Spain (Basque Country, Catalonia, Madrid, Valencia)	Harm reduction centres	Mar – Jun 2020	Mar – Jun 2019	Very heterogeneous, in Apr 2020 drop to 0 for 4 of 5 centres (from 9.5 -211.9 in Apr 2019), in May and June 2020 both increase and decrease as compared to May and June 2019	Picchio et al[82]
**New diagnoses**						
Change in the number of new cases	China(Jiangsu)	data from Comprehensive response Information Management System for HIV/AIDS in China	1st quarter of 2020	1st quarter of 2019	-258 cases (-24%)	Shi et al[138]
	Japan	Data from quarterly reports from the National AIDS surveillance Committee	2nd quarter of 2020	2nd quarter of 2019	-35 cases (-12%)	Ejima et al[113]
	Taiwan	Data from Taiwan's Centre for Disease Control	Jan-Aug 2020	Jan-Aug 2019	-293 cases (-23%)	Chia et al[150]
Monthly median of new cases	Belgium (Liege)	Data from Liege University Hospital	Apr – May 2020	Jan 2019 – Mar 2020	2 vs 8 (baseline)	Darcis et al[141]
Monthly mean number of new cases	Italy (Brescia)	HIV patients (tertiary referral center for Infectious Diseases and HIV in Brescia)	Mar-Apr 2020	2019	2.5 vs 6.4 (baseline)	Quiros-Roldan et al[115]
Weekly mean of positive tests	Uganda	Data from PEPFAR, Uganda ministry of Health and partners	Apr 2020 (weeks 14-16)	Oct 2019-Mar 2020 (up to week 13)	1284 vs 3224(baseline) = -60% [reported is -37% decline in June 2020, week 23]	Bell et al[37]
Number of new cases	Australia(Melbourne)	Data from Melbourne	1st quarter of 2020	1st quarter of 2019	9 vs 17(baseline)	Chow et al[143]

		Sexual Health Centre	2nd quarter of 2020	2nd quarter of 2019	15 vs 19(baseline)	
	Croatia	Data from University Hospital for Infectious Diseases, Zagreb, treating all PLWH in Croatia	Mar – Jun 2020	Mar –Jun 2019 and 2018	12 vs 22(in 2019) or 28(in 2018)	Bogdanic et al[133]
	Greece	Information from an expert panel of 9 HIV clinicians	Mar-May 2020	Jan-Feb 2020	Approx. 50% drop	Lourida et al[151]
	Greece(Alexandroupolis)	Data from HIV Clinic of Alexandroupolis	1st semester 2020	1st semester 2019	13 vs 6 (baseline) (diagnoses in 2020 mainly after lockdown)	Panagopoulos et al[152]
**HIV positivity rates(number of new HIV diagnoses as a percentage of the number of HIV tests done)**						
	Japan	Data from quarterly reports from the National AIDS surveillance Committee	2nd quarter of 2020	2nd quarter of 2019	260/9584(2.7%) vs 295/35908(0.8%)	Ejima et al[113]*
	China(Jiangsu)	data from Comprehensive response Information Management System for HIV/AIDS in China	1st quarter of 2020	1st quarter of 2019	749/1875685 (0.04%) vs 1057/2495990 (0.04%)	Shi et al[138]
	Australia (Melbourne)	Data from Melbourne Sexual Health Centre	1st quarter of 2020	1st quarter of 2019	9/7083 (0.13%) vs 17/8569 (0.2%)	Chow et al[143]
			2nd quarter of 2020	2nd quarter of 2019	15/4187 (0.36%) vs 19/7798(0.24%)	
	Belgium (Liege)	Data from Liege University Hospital	Jan –Mar 2020	Jan-Mar 2019	24/2605(0.92%) vs 28/3198 (0.88%)	Darcis et al[141]
			Apr-May 2020	Apr-May 2019	4/803(0.50%) vs 18/1939(0.93%)	
	Kenya, Zimbabwe, Malawi	18 centres in Nairobi, 9 in Harare, 8 in Lilongwe	Mar-Aug 2020	Mar-Aug 2020	5181/112635 (4.6%) vs 8379/246453 (3.4%)	Union[75]**

*Calculated from the reported numbers on HIV case with and without AIDS diagnoses. **The absolute numbers are calculated from the percentages.

S1 Table V Overview of the impact of individual HIV service/care interruptions on HIV-incidence and mortality as reported by the modeling studies. From studies related to African countries[35, 36, 42, 43, 168], we report only on the common comparison in [168]. For comparison, one study [38] considered 50% disruption in services(ART initiation, ART engagement, HIV testing, PrEP uptake, syringe service programme, medication for opioid users) over 12 months starting in March 2020 only combined and observed increase in HIV incidence over 2020-2025 in the range 7-16% for six US cities.

	% increase in HIV-incidence				% increase in HIV-related mortality		
	1-year incidence			5-year incidence	1-year mortality		
	Jewell[168], 50% disruption over 6 months, sub-Saharan Africa	Booton[171], 50% disruption over 3 months, 4 cities in China	Mitchell[169], 50% disruption over 6 months, MSM in Baltimore	Jenness[170], 50% disruption for 18 months, MSM in Atlanta area	Jewell[168] , 50% disruption over 6 months, sub-Saharan Africa	Booton[171], 50% disruption over 3 months, 4 cities in China,	Mitchell[169], 50% disruption over 6 months MSM in Baltimore
ART interruption	1-16%, 126%				39-87%		
ART retention				9%			
Condom use	3-19%	19%	10%		0%	0.5%	0%
PMTCT	2-5%				1-3%		
HIV testing	1%	1.8%	1%		0-1%	0.1%	0.1%
HIV screening				3.2%			
Viral load testing	0-3%				0-4%		
ART initiation	0-2%	2.4%	1.1%		0-3%	2.5%	5%
VMMC	0%				0%		
Increase in death rate due to overwhelmed health care	0%				2-6%		
Viral suppression		15%	29%			18%	46%
PrEP initiations				2.6%			

REFERENCES

1. Owolabi OA, Jallow AO, Jallow M, Sowe G, Jallow R, Genekah MD, et al. Delay in diagnosis of pulmonary tuberculosis in The Gambia, West Africa: a cross-sectional study. International journal of infectious diseases : IJID : official publication of the International Society for Infectious Diseases. 2020. Epub 2020/09/20. doi: 10.1016/j.ijid.2020.09.029. PubMed PMID: 32949776.

2. Riccardi N, Villa S, Canetti D, Giacomelli A, Taramasso L, Martini M, et al. Missed opportunities in tb clinical practice: How to bend the curve? A medical, social, economic and ethical point of view. Tuberculosis (Edinb). 2020;126:102041. Epub 2021/01/02. doi: 10.1016/j.tube.2020.102041. PubMed PMID: 33385833.

3. Mukwenha S, Dzinamarira T, Mugurungi O, Musuka G. Maintaining robust HIV and tuberculosis services in the COVID-19 era: A public health dilemma in Zimbabwe. International journal of infectious diseases : IJID : official publication of the International Society for Infectious Diseases. 2020;100:394-5. Epub 2020/09/27. doi: 10.1016/j.ijid.2020.09.1425. PubMed PMID: 32979586; PubMed Central PMCID: PMCPMC7513822.

4. Luis H, Fridayantara WD, Mahariski P, Wignall FS, Irwanto I, Gedela K. Evolving ART crisis for people living with HIV in Indonesia. The lancet HIV. 2020;7(6):e384-e5. Epub 2020/05/04. doi: 10.1016/s2352-3018(20)30138-7. PubMed PMID: 32359421; PubMed Central PMCID: PMCPMC7195127.

5. Kay ES, Musgrove K. From HIV to Coronavirus: AIDS Service Organizations Adaptative Responses to COVID-19, Birmingham, Alabama. AIDS and behavior. 2020;24(9):2461-2. Epub 2020/04/26. doi: 10.1007/s10461-020-02879-1. PubMed PMID: 32333206; PubMed Central PMCID: PMCPMC7180637.

6. Rozanova J, Shenoi S, Zaviryukha I, Zeziulin O, Kiriazova T, Rich K, et al. Social Support is Key to Retention in Care during Covid-19 Pandemic among Older People with HIV and Substance Use Disorders in Ukraine. Substance use & misuse. 2020;55(11):1902-4. Epub 2020/07/16. doi: 10.1080/10826084.2020.1791183. PubMed PMID: 32666857.

7. Fusco FM, Sangiovanni V, Tiberio C, Papa N, Atripaldi L, Esposito V. Persons living with HIV may be reluctant to access to COVID-19 testing services: data from "D. Cotugno" hospital, naples, southern Italy. Aids. 2020. Epub 2020/09/11. doi: 10.1097/qad.0000000000002678. PubMed PMID: 32910068.

8. Wang H. HIV care during the coronavirus disease-2019 pandemic in Shenzhen, China. Current opinion in HIV and AIDS. 2020. Epub 2020/09/25. doi: 10.1097/coh.0000000000000653. PubMed PMID: 32969977.

9. Sun S, Hou J, Chen Y, Lu Y, Brown L, Operario D. Challenges to HIV Care and Psychological Health During the COVID-19 Pandemic Among People Living with HIV in China. AIDS and behavior. 2020;24(10):2764-5. Epub 2020/05/10. doi: 10.1007/s10461-020-02903-4. PubMed PMID: 32382824; PubMed Central PMCID: PMCPMC7203543.

10. Ballivian J, Alcaide ML, Cecchini D, Jones DL, Abbamonte JM, Cassetti I. Impact of COVID-19-related stress and lockdown on mental health among people living with HIV in Argentina. J Acquir Immune Defic Syndr. 2020. Epub 2020/09/09. doi: 10.1097/qai.0000000000002493. PubMed PMID: 32897937.

11. Rhodes SD, Mann-Jackson L, Alonzo J, Garcia M, Tanner AE, Smart BD, et al. A Rapid Qualitative Assessment of the Impact of the COVID-19 Pandemic on a Racially/Ethnically Diverse Sample of Gay, Bisexual, and Other Men who Have Sex with Men Living with HIV in the US South. AIDS and behavior. 2020:1-10. Epub 2020/08/25. doi: 10.1007/s10461-020-03014-w. PubMed PMID: 32830296; PubMed Central PMCID: PMCPMC7443372.

12. Algarin AB, Varas-Rodríguez E, Valdivia C, Fennie KP, Larkey L, Hu N, et al. Symptoms, Stress, and HIV-Related Care Among Older People Living with HIV During the COVID-19 Pandemic, Miami, Florida. AIDS and behavior. 2020;24(8):2236-8. Epub 2020/04/19. doi: 10.1007/s10461-020-02869-3. PubMed PMID: 32303923; PubMed Central PMCID: PMCPMC7164703.

13. MacCarthy S, Izenberg M, Barreras JL, Brooks RA, Gonzalez A, Linnemayr S. Rapid mixed-methods assessment of COVID-19 impact on Latinx sexual minority men and Latinx transgender women. PLoS One. 2020;15(12):e0244421. Epub 2021/01/01. doi: 10.1371/journal.pone.0244421. PubMed PMID: 33382752; PubMed Central PMCID: PMCPMC7774921.
14. Ponticiello M, Mwanga-Amumpaire J, Tushemereirwe P, Nuwagaba G, King R, Sundararajan R. "Everything is a Mess": How COVID-19 is Impacting Engagement with HIV Testing Services in Rural Southwestern Uganda. AIDS and behavior. 2020;24(11):3006-9. Epub 2020/05/27. doi: 10.1007/s10461-020-02935-w. PubMed PMID: 32451939; PubMed Central PMCID: PMCPMC7246959.
15. Ridgway JP, Schmitt J, Friedman E, Taylor M, Devlin S, McNulty M, et al. HIV Care Continuum and COVID-19 Outcomes Among People Living with HIV During the COVID-19 Pandemic, Chicago, IL. AIDS and behavior. 2020;24(10):2770-2. Epub 2020/05/10. doi: 10.1007/s10461-020-02905-2. PubMed PMID: 32382823; PubMed Central PMCID: PMCPMC7203502.
16. Pierre G, Uwineza A, Dzinamarira T. Attendance to HIV Antiretroviral Collection Clinic Appointments During COVID-19 Lockdown. A Single Center Study in Kigali, Rwanda. AIDS and behavior. 2020:1-3. Epub 2020/06/27. doi: 10.1007/s10461-020-02956-5. PubMed PMID: 32588258; PubMed Central PMCID: PMCPMC7315689.
17. Giuliani M, Dona MG, La Malfa A, Pasquantonio MS, Pimpinelli F, Cristaudo A, et al. Ensuring retention in care for people living with HIV during the COVID-19 pandemic in Rome, Italy. Sex Transm Infect. 2020. Epub 2020/10/14. doi: 10.1136/sextrans-2020-054650. PubMed PMID: 33046581; PubMed Central PMCID: PMCPMC7551733.
18. Amatavete S, Seekaew P, Lujintanon S, Chinbunchorn T, Teeratakulpisarn N, Teeratakulpisarn S, et al. Telehealth for the continuation of same-day antiretroviral therapy during the COVID-19 outbreak and beyond. AIDS 2020 Conference, 2020.
19. Dakshina S, Orkin C, Dragovic B, Umaipalan A, Apea V. The impact of the COVID-19 pandemic on patient re-engagement within five London HIV centres. AIDS 2020 Conference,2020.
20. Jarolimova J, Bunda B, Govere S, Wara N, Bogart L, Thulare H, et al. Expperiences of participants in a decentralized HIV medication distribution program in South Africa during the COVID-19 pandemic. AIDS 2020 Conference, 2020.
21. Guaraldi G, Milic J, Ciusa G, Raimondi A, Cuomo G, Carli F, et al. Resilience and frailty in people living with HIV during lockdown experience in Italy in March-May 2020: are they two complementary constructs? Abstract Supplement HIV Glasgow - Virtual, 5-8 October 2020, J of Int AIDS Society. 2020;23:110. doi: 10.1002/jjia2.25616.
22. Lyons N, Edwards J. Using differentiated models of care to maintain gains in ART retention during the COVID-19 pandemic: Lessons from a large treatment facility in Trinidad and Tobago. AIDS 2020 Conference, 2020.
23. EATG Rapid Assessment 1. COVID-19 crisis' Impact on PLHIV and on Communities Most Affected by HIV. European AIDS Treatment Group, 2020.
24. EATG Rapid Assessment 2. COVID-19 crisis' Impact on PLHIV and on Communities Most Affected by HIV. European AIDS Treatment Group, 2020.
25. Impact of the COVID-19 Pandemic. Report of the findings of the impact of SARS-CoV-2-2019/COVID-19 pandemic on member organisations within AIDS Action Europe network. AIDS Action Europe, 2020.
26. Hussein N. The impact of covid-19 pandemic on the elimination of viral hepatitis in duhok city, kurdistan region of iraq. Hepat Mon. 2020;20(5). doi: 10.5812/hepatmon.104643.
27. Wingrove C, Ferrier L, James C, Wang S. The impact of COVID-19 on hepatitis elimination. The lancet Gastroenterology & hepatology. 2020;5(9):792-4. Epub 2020/07/31. doi: 10.1016/s2468-1253(20)30238-7. PubMed PMID: 32730783; PubMed Central PMCID: PMCPMC7384773.
28. Aikpon R, Affoukou C, Hounpkatin B, Eclou DD, Cyaka Y, Egwu E, et al. Digitalized mass distribution campaign of insecticide-treated nets (ITNs) in the particular context of Covid-19

pandemic in Benin: challenges and lessons learned. Malar J. 2020;19(1):431. Epub 2020/11/27. doi: 10.1186/s12936-020-03508-x. PubMed PMID: 33239004; PubMed Central PMCID: PMCPMC7686833.
29. Nussbaumer-Streit B, Mayr V, Dobrescu AI, Chapman A, Persad E, Klerings I, et al. Quarantine alone or in combination with other public health measures to control COVID-19: a rapid review. Cochrane Database Syst Rev. 2020;9:CD013574. Epub 2021/05/08. doi: 10.1002/14651858.CD013574.pub2. PubMed PMID: 33959956; PubMed Central PMCID: PMCPMC8133397.
30. Pitman R, Fisman D, Zaric GS, Postma M, Kretzschmar M, Edmunds J, et al. Dynamic transmission modeling: a report of the ISPOR-SMDM Modeling Good Research Practices Task Force--5. Value Health. 2012;15(6):828-34. Epub 2012/09/25. doi: 10.1016/j.jval.2012.06.011. PubMed PMID: 22999132; PubMed Central PMCID: PMCPMC7110742.
31. Risk of Bias Instrument for Cross-Sectional Surveys of Attitudes and Practices. CLARITY Group at McMaster University, 2021.
32. Burns KEA, Kho ME. How to assess a survey report: a guide for readers and peer reviewers. CMAJ. 2015;187(6):E198-E205. Epub 2015/02/19. doi: 10.1503/cmaj.140545. PubMed PMID: 25691790; PubMed Central PMCID: PMCPMC4387061.
33. Santesso N, Akl E, Bhandari M, Busse JW, Cook DJ, Greenhalgh T, et al. A practical guide for using a survey about attitudes and behaviors to inform health care decisions. J Clin Epidemiol. 2020;128:93-100. Epub 2020/09/29. doi: 10.1016/j.jclinepi.2019.11.020. PubMed PMID: 32987165.
34. Methods for the development of NICE public health guidance(third edition). National Institute for Health and Care Excellence, 2012.
35. Hogan AB, Jewell BL, Sherrard-Smith E, Vesga JF, Watson OJ, Whittaker C, et al. Potential impact of the COVID-19 pandemic on HIV, tuberculosis, and malaria in low-income and middle-income countries: a modelling study. The Lancet Global health. 2020;8(9):e1132-e41. Epub 2020/07/17. doi: 10.1016/s2214-109x(20)30288-6. PubMed PMID: 32673577; PubMed Central PMCID: PMCPMC7357988.
36. Stover J, Chagoma N, Taramusi I, Teng Y, Glaubius R, Mahiane SG. Estimation of the Potential Impact of COVID-19 Responses on the HIV Epidemic: Analysis using the Goals Model. 2020. doi: 10.1101/2020.05.04.20090399 %J medrxiv.
37. Bell D, Hansen KS, Kiragga AN, Kambugu A, Kissa J, Mbonye AK. Predicting the Impact of COVID-19 and the Potential Impact of the Public Health Response on Disease Burden in Uganda. The American journal of tropical medicine and hygiene. 2020;103(3):1191-7. Epub 2020/07/25. doi: 10.4269/ajtmh.20-0546. PubMed PMID: 32705975; PubMed Central PMCID: PMCPMC7470592.
38. Zang X, Krebs E, Chen S, Piske M, Armstrong WS, Behrends CN, et al. The potential epidemiological impact of COVID-19 on the HIV/AIDS epidemic and the cost-effectiveness of linked, opt-out HIV testing: A modeling study in six US cities. Clinical infectious diseases : an official publication of the Infectious Diseases Society of America. 2020. Epub 2020/10/13. doi: 10.1093/cid/ciaa1547. PubMed PMID: 33045723; PubMed Central PMCID: PMCPMC7665350.
39. Roberton T, Carter ED, Chou VB, Stegmuller AR, Jackson BD, Tam Y, et al. Early estimates of the indirect effects of the COVID-19 pandemic on maternal and child mortality in low-income and middle-income countries: a modelling study. The Lancet Global Health. 2020;8(7):e901-e8. doi: 10.1016/s2214-109x(20)30229-1.
40. Glaziou P. Predicted impact of the COVID-19 pandemic on global tuberculosis deaths in 2020. 2020. doi: 10.1101/2020.04.28.20079582 %J medrxiv.
41. Lai AG, Pasea L, Banerjee A, Hall G, Denaxas S, Chang WH, et al. Estimated impact of the COVID-19 pandemic on cancer services and excess 1-year mortality in people with cancer and multimorbidity: near real-time data on cancer care, cancer deaths and a population-based cohort study. BMJ Open. 2020;10(11):e043828. Epub 2020/11/19. doi: 10.1136/bmjopen-2020-043828. PubMed PMID: 33203640; PubMed Central PMCID: PMCPMC7674020.

42. Jewell BL, Smith JA, Hallett TB. The Potential Impact of Interruptions to HIV Services: A Modelling Case Study for South Africa. 2020. doi: 10.1101/2020.04.22.20075861 %J medrxiv.
43. Jewell BL, Smith JA, Hallett TB. Understanding the impact of interruptions to HIV services during the COVID-19 pandemic: A modelling study. EClinicalMedicine. 2020. doi: 10.1016/j.eclinm.2020.100483.
44. Louie JK, Reid M, Stella J, Agraz-Lara R, Graves S, Chen L, et al. A decrease in tuberculosis evaluations and diagnoses during the COVID-19 pandemic. Int J Tuberc Lung Dis. 2020;24(8):860-2. Epub 2020/09/12. doi: 10.5588/ijtld.20.0364. PubMed PMID: 32912395.
45. Chandir S, Siddiqi DA, Mehmood M, Setayesh H, Siddique M, Mirza A, et al. Impact of COVID-19 pandemic response on uptake of routine immunizations in Sindh, Pakistan: An analysis of provincial electronic immunization registry data. Vaccine. 2020;38(45):7146-55. Epub 2020/09/19. doi: 10.1016/j.vaccine.2020.08.019. PubMed PMID: 32943265; PubMed Central PMCID: PMCPMC7428732.
46. Shrinivasan R, Rane S, Pai M. India's syndemic of tuberculosis and COVID-19. BMJ global health. 2020;5(11). Epub 2020/11/18. doi: 10.1136/bmjgh-2020-003979. PubMed PMID: 33199280; PubMed Central PMCID: PMCPMC7670552.
47. Wu Z, Chen J, Xia Z, Pan Q, Yuan Z, Zhang W, et al. Impact of the COVID-19 pandemic on the detection of TB in Shanghai, China. Int J Tuberc Lung Dis. 2020;24(10):1122-4. Epub 2020/11/01. doi: 10.5588/ijtld.20.0539. PubMed PMID: 33126952.
48. Migliori GB, Thong PM, Akkerman O, Alffenaar JW, Álvarez-Navascués F, Assao-Neino MM, et al. Worldwide Effects of Coronavirus Disease Pandemic on Tuberculosis Services, January-April 2020. Emerging infectious diseases. 2020;26(11). Epub 2020/09/13. doi: 10.3201/eid2611.203163. PubMed PMID: 32917293.
49. Cronin AM, Railey S, Fortune D, Wegener DH, Davis JB. Notes from the Field: Effects of the COVID-19 Response on Tuberculosis Prevention and Control Efforts - United States, March-April 2020. MMWR Morbidity and mortality weekly report. 2020;69(29):971-2. Epub 2020/07/24. doi: 10.15585/mmwr.mm6929a4. PubMed PMID: 32701944; PubMed Central PMCID: PMCPMC7377818
50. Khan MS, Rego S, Rajal JB, Bond V, Fatima RK, Isani AK, et al. Mitigating the impact of COVID-19 on tuberculosis and HIV services: a cross-sectional survey of 669 health professionals in 64 low and middle-income countries. medRxiv. 2020. doi: 10.1101/2020.10.08.20207969.
51. Nikolayevskyy V, Holicka Y, van Soolingen D, van der Werf MJ, Kodmon C, Surkova E, et al. Impact of the COVID-19 pandemic on tuberculosis laboratory services in Europe. The European respiratory journal. 2021;57(1). Epub 2020/11/14. doi: 10.1183/13993003.03890-2020. PubMed PMID: 33184119; PubMed Central PMCID: PMCPMC7670866.
52. Shen X, Sha W, Yang C, Pan Q, Cohen T, Cheng S, et al. Continuity of TB services during the COVID-19 pandemic in China. Int J Tuberc Lung Dis. 2021;25(1):81-3. Epub 2021/01/02. doi: 10.5588/ijtld.20.0632. PubMed PMID: 33384053.
53. Udwadia ZF, Sharma S, Mullerpattan JB, Gajjar I, Pinto L. Effective use of telemedicine in Mumbai with a cohort of extensively drug-resistant "XDR" tuberculosis patients on bedaquiline during COVID-19 pandemic. Lung India. 2021;38(1):98-9. Epub 2021/01/07. doi: 10.4103/lungindia.lungindia_464_20. PubMed PMID: 33402651.
54. Mitigating the impact of Covid-19 on countries affected by HIV, tuberculosis and malaria. Geneva: The Global Fund to Fight AIDS, Tuberculosis and Malaria, 2020.
55. Global Tuberculosis Report 2020. Geneva: World Health Organization; 2020.
56. Behera D. TB control in India in the COVID era. Indian J Tuberc. 2020. doi: 10.1016/j.ijtb.2020.08.019.
57. Bhargava A, Shewade HD. The potential impact of the COVID-19 response related lockdown on TB incidence and mortality in India. Indian J Tuberc. 2020. doi: 10.1016/j.ijtb.2020.07.004.
58. Chen H, Zhang K. Insight into the impact of the COVID-19 epidemic on tuberculosis burden in China. The European respiratory journal. 2020;56(3). Epub 2020/07/25. doi:

10.1183/13993003.02710-2020. PubMed PMID: 32703778; PubMed Central PMCID: PMCPMC7397949.
59. Chiang CY, Islam T, Xu C, Chinnayah T, Garfin AMC, Rahevar K, et al. The impact of COVID-19 and the restoration of tuberculosis services in the Western Pacific Region (revised). The European respiratory journal. 2020. Epub 2020/09/27. doi: 10.1183/13993003.03054-2020. PubMed PMID: 32978310.
60. Coronel Teixeira R, Aguirre S, Perez Bejarano D. Thinking about tuberculosis in times of COVID-19. J Intern Med. 2020. Epub 2020/10/21. doi: 10.1111/joim.13192. PubMed PMID: 33078450.
61. Fang JL, Chao CM, Tang HJ. The impact of COVID-19 on the diagnosis of TB in Taiwan. Int J Tuberc Lung Dis. 2020;24(12):1321-2. Epub 2020/12/16. doi: 10.5588/ijtld.20.0746. PubMed PMID: 33317682.
62. Iyengar KP, Jain VK. Tuberculosis and COVID-19 in India- double trouble! Indian J Tuberc. 2020. doi: 10.1016/j.ijtb.2020.07.014.
63. Kwak N, Hwang SS, Yim JJ. Effect of COVID-19 on Tuberculosis Notification, South Korea. Emerging infectious diseases. 2020;26(10):2506-8. Epub 2020/07/17. doi: 10.3201/eid2610.202782. PubMed PMID: 32672531.
64. Komiya K, Yamasue M, Takahashi O, Hiramatsu K, Kadota JI, Kato S. The COVID-19 pandemic and the true incidence of Tuberculosis in Japan. The Journal of infection. 2020;81(3):e24-e5. Epub 2020/07/11. doi: 10.1016/j.jinf.2020.07.004. PubMed PMID: 32650109; PubMed Central PMCID: PMCPMC7338857.
65. Lai CC, Yu WL. The COVID-19 pandemic and tuberculosis in Taiwan. The Journal of infection. 2020;81(2):e159-e61. Epub 2020/06/14. doi: 10.1016/j.jinf.2020.06.014. PubMed PMID: 32534000; PubMed Central PMCID: PMCPMC7286835.
66. Liu Q, Lu P, Shen Y, Li C, Wang J, Zhu L, et al. Collateral Impact of the Covid-19 Pandemic on Tuberculosis Control in Jiangsu Province, China. Clinical infectious diseases : an official publication of the Infectious Diseases Society of America. 2020. Epub 2020/08/29. doi: 10.1093/cid/ciaa1289. PubMed PMID: 32857838; PubMed Central PMCID: PMCPMC7499510.
67. Mohammed H, Oljira L, Roba KT, Yimer G, Fekadu A, Manyazewal T. Containment of COVID-19 in Ethiopia and implications for tuberculosis care and research. Infectious diseases of poverty. 2020;9(1):131. Epub 2020/09/18. doi: 10.1186/s40249-020-00753-9. PubMed PMID: 32938497.
68. de Souza CDF, Coutinho HS, Costa MM, Magalhaes M, Carmo RF. Impact of COVID-19 on TB diagnosis in Northeastern Brazil. Int J Tuberc Lung Dis. 2020;24(11):1220-2. Epub 2020/11/12. doi: 10.5588/ijtld.20.0661. PubMed PMID: 33172534.
69. Buonsenso D, Iodice F, Sorba Biala J, Goletti D. COVID-19 effects on tuberculosis care in Sierra Leone. Pulmonology. 2020. Epub 2020/06/21. doi: 10.1016/j.pulmoe.2020.05.013. PubMed PMID: 32561353; PubMed Central PMCID: PMCPMC7275172.
70. Datta B, Jaiswal A, Goyal P, Prakash A, Tripathy JP, Trehan N. The untimely demise of the TB Free block model in the wake of coronavirus disease 2019 in India. Trans R Soc Trop Med Hyg. 2020;114(11):789-91. Epub 2020/08/17. doi: 10.1093/trstmh/traa067. PubMed PMID: 32797204; PubMed Central PMCID: PMCPMC7454845.
71. Magro P, Formenti B, Marchese V, Gulletta M, Tomasoni LR, Caligaris S, et al. Impact of the SARS Coronavirus 2 epidemic on tuberculosis treatment outcome, Northern Italy. The European respiratory journal. 2020. Epub 2020/07/25. doi: 10.1183/13993003.02665-2020. PubMed PMID: 32703780; PubMed Central PMCID: PMCPMC7377210.
72. Kadota JL, Reza TF, Nalugwa T, Kityamuwesi A, Nanyunja G, Kiwanuka N, et al. Impact of shelter-in-place on TB case notifications and mortality during the COVID-19 pandemic. Int J Tuberc Lung Dis. 2020;24(11):1212-4. Epub 2020/11/12. doi: 10.5588/ijtld.20.0626. PubMed PMID: 33172531.
73. Lebina L, Dube M, Hlongwane K, Brahmbatt H, Lala SG, Reubenson G, et al. Trends in paediatric tuberculosis diagnoses in two South African hospitals early in the COVID-19 pandemic.

S Afr Med J. 2020;110(12):1149-50. Epub 2021/01/07. doi: 10.7196/SAMJ.2020.v110i12.15386. PubMed PMID: 33403952.
74. Min J, Kim HW, Koo HK, Ko Y, Oh JY, Kim J, et al. Impact of COVID-19 Pandemic on the National PPM Tuberculosis Control Project in Korea: the Korean PPM Monitoring Database between July 2019 and June 2020. J Korean Med Sci. 2020;35(43):e388. Epub 2020/11/11. doi: 10.3346/jkms.2020.35.e388. PubMed PMID: 33169559; PubMed Central PMCID: PMCPMC7653169.
75. Mid-term report on the impact of COVID-19 on TB and HIV in Africa. 2020.
76. Cilloni L, Fu H, Vesga JF, Dowdy D, Pretorius C, Ahmedov S, et al. The potential impact of the COVID-19 pandemic on the tuberculosis epidemic a modelling analysis. EClinicalMedicine. 2020;28:100603. Epub 2020/11/03. doi: 10.1016/j.eclinm.2020.100603. PubMed PMID: 33134905; PubMed Central PMCID: PMCPMC7584493.
77. McQuaid CF, McCreesh N, Read JM, Sumner T, Houben R, White RG, et al. The potential impact of COVID-19-related disruption on tuberculosis burden. The European respiratory journal. 2020;56(2). Epub 2020/06/10. doi: 10.1183/13993003.01718-2020. PubMed PMID: 32513784; PubMed Central PMCID: PMCPMC7278504.
78. The Potential Impact of the COVID-19 Response on Tuberculosis in High-Burden Countries: a Modelling Study. Stop TB Partnership, 2020.
79. Simoes D, Stengaard AR, Combs L, Raben D, Euro TC-iacop. Impact of the COVID-19 pandemic on testing services for HIV, viral hepatitis and sexually transmitted infections in the WHO European Region, March to August 2020. Euro Surveill. 2020;25(47). Epub 2020/11/28. doi: 10.2807/1560-7917.ES.2020.25.47.2001943. PubMed PMID: 33243354; PubMed Central PMCID: PMCPMC7693166.
80. Huppe D, Niederau C, Serfert Y, Hartmann H, Wedemeyer H, fur das D-R. [Problems in treating patients with chronic HCV infection due to the COVID-19 pandemic and during the lockdown phase in Germany]. Z Gastroenterol. 2020;58(12):1182-5. Epub 2020/11/10. doi: 10.1055/a-1291-8518. PubMed PMID: 33167051; PubMed Central PMCID: PMCPMC7724581 Vortragshonorare, Forschungsgelder oder Beraterhonorare) erhalten von: AbbVie GmbH & Co. KG, Falk Pharma, Ferring Arzneimittel GmbH. Prof. Dr. Claus Niederau hat in den letzten 3 Jahren Zuwendungen (z. B. Vortragshonorare, Forschungsgelder oder Beraterhonorare) erhalten von: Abbvie, Alexion, Biogen, BMS, Falk, Gilead, Janssen, MSD, Sanofi-Genzyme und Shire-Takeda. Dr. Yvonne Serfert hat keine Interessenkonflikte. Prof. Dr. Heinz Hartmann hat in den letzten 3 Jahren Zuwendungen (Vortragshonorare) erhalten von: Falk Foundation. Prof. Dr. Heiner Wedemeyer hat in den letzten 3 Jahren Zuwendungen (z. B. Vortragshonorare, Forschungsgelder oder Beraterhonorare) erhalten von: Abbott, AbbVie, Altimmune, Biotest, BMS, BTG, Dicerna, Gilead, Janssen, Merck/MSD, MYR GmbH, Novartis, Roche, Siemens, Transgene.
81. Aghemo A, Masarone M, Montagnese S, Petta S, Ponziani FR, Russo FP. Assessing the impact of COVID-19 on the management of patients with liver diseases: A national survey by the Italian association for the study of the Liver. Digestive and liver disease : official journal of the Italian Society of Gastroenterology and the Italian Association for the Study of the Liver. 2020;52(9):937-41. Epub 2020/07/25. doi: 10.1016/j.dld.2020.07.008. PubMed PMID: 32703730; PubMed Central PMCID: PMCPMC7351426.
82. Picchio CA, Valencia J, Doran J, Swan T, Pastor M, Martro E, et al. The impact of the COVID-19 pandemic on harm reduction services in Spain. Harm Reduct J. 2020;17(1):87. Epub 2020/11/05. doi: 10.1186/s12954-020-00432-w. PubMed PMID: 33143699; PubMed Central PMCID: PMCPMC7609370.
83. Lemoine M, Kim JU, Ndow G, Bah S, Forrest K, Rwegasha J, et al. Effect of the COVID-19 pandemic on viral hepatitis services in sub-Saharan Africa. The lancet Gastroenterology & hepatology. 2020. Epub 2020/09/21. doi: 10.1016/s2468-1253(20)30305-8. PubMed PMID: 32950107.
84. Blach S, Kondili LA, Aghemo A, Cai Z, Dugan E, Estes C, et al. Impact of COVID-19 on global hepatitis C elimination efforts. Journal of hepatology. 2020. Epub 2020/08/11. doi:

10.1016/j.jhep.2020.07.042. PubMed PMID: 32777322; PubMed Central PMCID: PMCPMC7411379.
85. Abbas K, Procter SR, van Zandvoort K, Clark A, Funk S, Mengistu T, et al. Routine childhood immunisation during the COVID-19 pandemic in Africa: a benefit-risk analysis of health benefits versus excess risk of SARS-CoV-2 infection. The Lancet Global health. 2020;8(10):e1264-e72. Epub 2020/07/21. doi: 10.1016/s2214-109x(20)30308-9. PubMed PMID: 32687792; PubMed Central PMCID: PMCPMC7367673.
86. Amaddeo G, Brustia R, Allaire M, Lequoy M, Hollande C, Regnault H, et al. Impact of COVID-19 on the management of hepatocellular carcinoma in a high-prevalence area. JHEP Rep. 2021;3(1):100199. Epub 2020/11/10. doi: 10.1016/j.jhepr.2020.100199. PubMed PMID: 33163949; PubMed Central PMCID: PMCPMC7604130.
87. Yun HE, Ryu BY, Choe YJ. Impact of social distancing on incidence of vaccine-preventable diseases, South Korea. J Med Virol. 2020. Epub 2020/10/21. doi: 10.1002/jmv.26614. PubMed PMID: 33079384.
88. Steffen R, Lautenschlager S, Fehr J. Travel restrictions and lockdown during the COVID-19 pandemic-impact on notified infectious diseases in Switzerland. J Travel Med. 2020;27(8). Epub 2020/11/06. doi: 10.1093/jtm/taaa180. PubMed PMID: 33152761; PubMed Central PMCID: PMCPMC7543597.
89. Eshraghian A, Taghavi A, Nikeghbalian S, Malek-Hosseini SA. Reduced rate of hospital admissions for liver-related morbidities during the initial COVID-19 outbreak. Lancet Gastroenterol Hepatol. 2020;5(9):803-4. doi: 10.1016/S2468-1253(20)30207-7. PubMed Central PMCID: PMC32615070.
90. Bramer CA, Kimmins LM, Swanson R, Kuo J, Vranesich P, Jacques-Carroll LA, et al. Decline in child vaccination coverage during the COVID-19 pandemic — Michigan Care Improvement Registry, May 2016-May 2020. Am J Transplant. 2020;20(7):1930-1. doi: 10.1111/ajt.16112. PubMed Central PMCID: PMC32437340.
91. Gaspar R, Liberal R, Branco CC, Macedo G. Trends in cirrhosis hospitalizations during the COVID-19 pandemic. Dig Liver Dis. 2020;52(9):942-3. doi: 10.1016/j.dld.2020.06.044. PubMed Central PMCID: PMC32680758.
92. McDonald HI, Tessier E, White JM, Woodruff M, Knowles C, Bates C, et al. Early impact of the coronavirus disease (COVID-19) pandemic and physical distancing measures on routine childhood vaccinations in England, January to April 2020. Eurosurveillance. 2020;25(19). doi: 10.2807/1560-7917.ES.2020.25.19.2000848. PubMed Central PMCID: PMC32431288.
93. Karimi-Sari H, Rezaee-Zavareh MS. COVID-19 and viral hepatitis elimination programs: Are we stepping backward? Liver international : official journal of the International Association for the Study of the Liver. 2020. Epub 2020/04/23. doi: 10.1111/liv.14486. PubMed PMID: 32319207.
94. Toyoda H, Yasuda S, Kiriyama S, Tanikawa M, Hisanaga Y, Kanamori A, et al. Impact of COVID-19 pandemic on surveillance of hepatocellular carcinoma: a study in patients with chronic hepatitis C after sustained virologic response. GastroHep. 2020. Epub 2020/08/25. doi: 10.1002/ygh2.418. PubMed PMID: 32837333; PubMed Central PMCID: PMCPMC7436720.
95. Toyoda H, Huang DQ, Le MH, Nguyen MH. Liver Care and Surveillance: The Global Impact of the COVID-19 Pandemic. Hepatology communications. 2020. Epub 2020/08/25. doi: 10.1002/hep4.1579. PubMed PMID: 32838107; PubMed Central PMCID: PMCPMC7405084.
96. Téllez L, Martín Mateos RM. COVID-19 and liver disease: An update. Gastroenterologia y hepatologia. 2020. Epub 2020/07/31. doi: 10.1016/j.gastrohep.2020.06.006. PubMed PMID: 32727662; PubMed Central PMCID: PMCPMC7332955.
97. 19.4% drop in Hepatitis-B birth doses, 31% drop in vaccination sessions in April-June [Internet]. 2020 [cited September 30, 2020]. Available from: https://hepvoices.org/2020/09/19-4-drop-in-hepatitis-b-birth-doses-31-drop-in-vaccination-sessions-in-april-june/
98. Sherrard-Smith E, Hogan AB, Hamlet A, Watson OJ, Whittaker C, Winskill P, et al. The potential public health consequences of COVID-19 on malaria in Africa. Nature medicine. 2020;26(9):1411-6. Epub 2020/08/10. doi: 10.1038/s41591-020-1025-y. PubMed PMID: 32770167.

99. Shi B, Zheng J, Xia S, Lin S, Wang X, Liu Y, et al. Accessing the syndemic of COVID-19 and malaria intervention in Africa. Infectious diseases of poverty. 2021;10(1):5. Epub 2021/01/09. doi: 10.1186/s40249-020-00788-y. PubMed PMID: 33413680; PubMed Central PMCID: PMCPMC7788178.
100. Weiss DJ, Bertozzi-Villa A, Rumisha SF, Amratia P, Arambepola R, Battle KE, et al. Indirect effects of the COVID-19 pandemic on malaria intervention coverage, morbidity, and mortality in Africa: a geospatial modelling analysis. The Lancet Infectious diseases. 2020. Epub 2020/09/25. doi: 10.1016/s1473-3099(20)30700-3. PubMed PMID: 32971006.
101. The potential impact of health service disruptions on the burden of malaria: a modelling analysis for countries in sub-Saharan Africa. Geneva: World Health Organization; 2020.
102. Buonsenso D, Cinicola B, Kallon MN, Iodice F. Child Healthcare and Immunizations in Sub-Saharan Africa During the COVID-19 Pandemic. Frontiers in pediatrics. 2020;8:517. Epub 2020/08/28. doi: 10.3389/fped.2020.00517. PubMed PMID: 32850565; PubMed Central PMCID: PMCPMC7424001.
103. Torres K, Alava F, Soto-Calle V, Llanos-Cuentas A, Rodriguez H, Llacsahuanga L, et al. Malaria Situation in the Peruvian Amazon during the COVID-19 Pandemic. The American journal of tropical medicine and hygiene. 2020. Epub 2020/09/05. doi: 10.4269/ajtmh.20-0889. PubMed PMID: 32885776.
104. Guerra CA, Tresor Donfack O, Motobe Vaz L, Mba Nlang JA, Nze Nchama LO, Mba Eyono JN, et al. Malaria vector control in sub-Saharan Africa in the time of COVID-19: no room for complacency. BMJ global health. 2020;5(9). Epub 2020/09/18. doi: 10.1136/bmjgh-2020-003880. PubMed PMID: 32938611.
105. Feldman M, Vernaeve L, Tibenderana J, Debackere M, Braack L, Thu HK, et al. The importance of building community trust for sustained health interventions during disruptive events such as COVID-19: A Cambodia case study. ResearchSquare. 2020. doi: 10.21203/rs.3.rs-94629/v1.
106. Buonsenso D, Iodice F, Cinicola B, Raffaelli F, Sowa S, Ricciardi W. Management of malaria in children under 5-years-old during COVID-19 pandemic in Sierra Leone: a lesson learned? medRxiv. 2020. doi: 10.1101/2020.11.04.20225714.
107. Penjor K, Tobgyal, Zangpo T, Clements ACA, Gray DJ, Wangdi K. Has COVID19 derailed Bhutan's national malaria elimination goal? A commentary. Malar J. 2021;20(1):20. Epub 2021/01/08. doi: 10.1186/s12936-020-03562-5. PubMed PMID: 33407471; PubMed Central PMCID: PMCPMC7787406.
108. Ranaweera P, Wickremasinghe R, Mendis K. Preventing the re-establishment of malaria in Sri Lanka amidst the COVID-19 pandemic. Malar J. 2020;19(1):386. Epub 2020/11/04. doi: 10.1186/s12936-020-03465-5. PubMed PMID: 33138814; PubMed Central PMCID: PMCPMC7605332.
109. Thapa B, Thi A, Than WP, Win KM, Khine SK. Myanmar Continues to Curb Malaria amid Coronavirus Disease-2019 Crisis. ResearchSquare. 2020. doi: 10.21203/rs.3.rs-101547/v1.
110. Emmanuel Awucha N, Chinelo Janefrances O, Chima Meshach A, Chiamaka Henrietta J, Ibilolia Daniel A, Esther Chidiebere N. Impact of the COVID-19 Pandemic on Consumers' Access to Essential Medicines in Nigeria. The American journal of tropical medicine and hygiene. 2020;103(4):1630-4. Epub 2020/08/21. doi: 10.4269/ajtmh.20-0838. PubMed PMID: 32815509; PubMed Central PMCID: PMCPMC7543821.
111. Best practices in mitigating the effect of COVID-19 on malaria. Geneva: RBM Partnership To End Malaria, 2021.
112. World malaria report 2020: 20 years of global progress and challenges. Geneva: World Health Organization; 2020.
113. Ejima K, Koizumi Y, Yamamoto N, Rosenberg M, Ludema C, Bento AI, et al. HIV testing by public health centers and municipalities, and new HIV cases during the COVID-19 pandemic in Japan. medRxiv. 2020. doi: 10.1101/2020.10.16.20213959.
114. Mitsuya H. Fight against COVID-19 but avoid disruption of services for other communicable diseases (CDs) and noncommunicable diseases (NCDs). Glob Health Med.

2020;2(6):343-5. Epub 2021/01/08. doi: 10.35772/ghm.2020.01111. PubMed PMID: 33409412; PubMed Central PMCID: PMCPMC7780288.
115. Quiros-Roldan E, Magro P, Carriero C, Chiesa A, El Hamad I, Tratta E, et al. Consequences of the COVID-19 pandemic on the continuum of care in a cohort of people living with HIV followed in a single center of Northern Italy. AIDS Res Ther. 2020;17(1):59. Epub 2020/10/06. doi: 10.1186/s12981-020-00314-y. PubMed PMID: 33012282; PubMed Central PMCID: PMCPMC7533114.
116. Spinelli MA, Hickey MD, Glidden DV, Nguyen JQ, Oskarsson JJ, Havlir D, et al. Viral suppression rates in a safety-net HIV clinic in San Francisco destabilized during COVID-19. Aids. 2020. Epub 2020/09/11. doi: 10.1097/qad.0000000000002677. PubMed PMID: 32910069.
117. Hickey MD, Imbert E, Glidden DV, Rosario JBD, Chong M, Clemenzi-Allen A, et al. Viral suppression not decreased during COVID-19 among people with HIV and unstable housing enrolled in a low-barrier clinic-based program (POP-UP). AIDS. 2020. Epub 2020/12/12. doi: 10.1097/QAD.0000000000002793. PubMed PMID: 33306555.
118. Madhi SA, Gray GE, Ismail N, Izu A, Mendelson M, Cassim N, et al. COVID-19 lockdowns in low- and middle-income countries: Success against COVID-19 at the price of greater costs. S Afr Med J. 2020;110(8):724-6. Epub 2020/09/04. PubMed PMID: 32880296.
119. Bogart LM, Ojikutu BO, Tyagi K, Klein DJ, Mutchler MG, Dong L, et al. COVID-19 Related Medical Mistrust, Health Impacts, and Potential Vaccine Hesitancy Among Black Americans Living With HIV. J Acquir Immune Defic Syndr. 2021;86(2):200-7. Epub 2020/11/17. doi: 10.1097/QAI.0000000000002570. PubMed PMID: 33196555.
120. Kalichman SC, Eaton LA, Berman M, Kalichman MO, Katner H, Sam SS, et al. Intersecting Pandemics: Impact of SARS-CoV-2 (COVID-19) Protective Behaviors on People Living With HIV, Atlanta, Georgia. J Acquir Immune Defic Syndr. 2020;85(1):66-72. Epub 2020/06/13. doi: 10.1097/qai.0000000000002414. PubMed PMID: 32530862; PubMed Central PMCID: PMCPMC7447002.
121. Hochstatter KR, Akhtar WZ, Dietz S, Pe-Romashko K, Gustafson DH, Shah DV, et al. Potential Influences of the COVID-19 Pandemic on Drug Use and HIV Care Among People Living with HIV and Substance Use Disorders: Experience from a Pilot mHealth Intervention. AIDS and behavior. 2020:1-6. Epub 2020/07/25. doi: 10.1007/s10461-020-02976-1. PubMed PMID: 32705370; PubMed Central PMCID: PMCPMC7376523.
122. Sanchez TH, Zlotorzynska M, Rai M, Baral SD. Characterizing the Impact of COVID-19 on Men Who Have Sex with Men Across the United States in April, 2020. AIDS and behavior. 2020;24(7):2024-32. Epub 2020/05/01. doi: 10.1007/s10461-020-02894-2. PubMed PMID: 32350773; PubMed Central PMCID: PMCPMC7189633.
123. Torres TS, Hoagland B, Bezerra DRB, Garner A, Jalil EM, Coelho LE, et al. Impact of COVID-19 Pandemic on Sexual Minority Populations in Brazil: An Analysis of Social/Racial Disparities in Maintaining Social Distancing and a Description of Sexual Behavior. AIDS and behavior. 2020:1-12. Epub 2020/08/02. doi: 10.1007/s10461-020-02984-1. PubMed PMID: 32737817; PubMed Central PMCID: PMCPMC7394046.
124. Dyer J, Wilson K, Badia J, Agot K, Neary J, Njuguna I, et al. The Psychosocial Effects of the COVID-19 Pandemic on Youth Living with HIV in Western Kenya. AIDS and behavior. 2020:1-5. Epub 2020/08/21. doi: 10.1007/s10461-020-03005-x. PubMed PMID: 32816193; PubMed Central PMCID: PMCPMC7438976.
125. Linnemayr S, Jennings Mayo-Wilson L, Saya U, Wagner Z, MacCarthy S, Walukaga S, et al. HIV Care Experiences During the COVID-19 Pandemic: Mixed-Methods Telephone Interviews with Clinic-Enrolled HIV-Infected Adults in Uganda. AIDS and behavior. 2020. Epub 2020/09/13. doi: 10.1007/s10461-020-03032-8. PubMed PMID: 32918641.
126. Sun Y, Li H, Luo G, Meng X, Guo W, Fitzpatrick T, et al. Antiretroviral treatment interruption among people living with HIV during COVID-19 outbreak in China: a nationwide cross-sectional study. J Int AIDS Soc. 2020;23(11):e25637. Epub 2020/11/29. doi: 10.1002/jia2.25637. PubMed PMID: 33247541; PubMed Central PMCID: PMCPMC7645858.

127. Guo W, Weng HL, Bai H, Liu J, Wei XN, Zhou K, et al. [Quick community survey on the impact of COVID-19 outbreak for the healthcare of people living with HIV]. Zhonghua liu xing bing xue za zhi = Zhonghua liuxingbingxue zazhi. 2020;41(5):662-6. Epub 2020/04/01. doi: 10.3760/cma.j.cn112338-20200314-00345. PubMed PMID: 32223840.
128. Santos GM, Ackerman B, Rao A, Wallach S, Ayala G, Lamontage E, et al. Economic, Mental Health, HIV Prevention and HIV Treatment Impacts of COVID-19 and the COVID-19 Response on a Global Sample of Cisgender Gay Men and Other Men Who Have Sex with Men. AIDS and behavior. 2020:1-11. Epub 2020/07/13. doi: 10.1007/s10461-020-02969-0. PubMed PMID: 32654021; PubMed Central PMCID: PMCPMC7352092.
129. Siewe Fodjo JN, Faria de Moura Villela E, Van Hees S, Tibério Dos Santos T, Vanholder P, Vanholder P, et al. Impact of the COVID-19 pandemic on the medical follow-up and psychosocial well-being of people living with HIV: A cross-sectional survey. J Acquir Immune Defic Syndr. 2020. Epub 2020/08/23. doi: 10.1097/qai.0000000000002468. PubMed PMID: 32826562.
130. Rao A, Rucinski K, Jarrett B, Ackerman B, Wallach S, Marcus J, et al. Potential interruptions in HIV prevention and treatment services for gay, bisexual, and other men who have sex with men associated with COVID-19. 2020. doi: 10.1101/2020.08.19.20178285 %J medRxiv.
131. Ogirima F, Azurunwa O, Batur S, Abe O, Egwumba P, Ayo I, et al. Surging through the COVID-19 pannndemic to retain patients on ART: Experiences using innovative approaches for ART delivery in Lagos state, Nigeria. AIDS 2020 Conference, 2020.
132. Tun NN, Hlaing MMM, Smithuis F. Adapting and overcoming to the challenges of HIV prevention and treatment activities under the threat of SARS-CoV-2 in Myanmar. AIDS 2020 Conference, 2020.
133. Bogdanic N, Zekan S, Romih Pintar V, Lukas D, Begovac J. The impact of COVID-19 epidemic on HIV care in Croatia. Abstract Supplement HIV Glasgow - Virtual, 5-8 October, 2020, Journal of the International AIDS Society. 2020;23:e25616.
134. Seizing the Moment. Global AIDS Update 2020. Geneva: UNAIDS, 2020.
135. Prevailing Against Pandemics by Putting people at the Centre. UNAIDS, 2020.
136. Pulse survey on continuity of essential health services during the COVID-19 pandemic. Interim report. Geneva: World health Organization, 2020.
137. Lamontagne E, Doan TT, Howell S, Yakusik A, Baral S, Strömdahl S, et al. COVID-19 pandemic increases socioeconomic vulnerabiility of LGBT+ communities and their susceptibility to HIV. AIDS 20202020.
138. Shi L, Tang W, Hu H, Qiu T, Marley G, Liu X, et al. Impact of the COVID-19 pandemic on HIV care continuum in Jiangsu, China. 2020. doi: 10.21203/rs.3.rs-135421/v1.
139. Jensen C, McKerrow NH. Child health services during a COVID-19 outbreak in KwaZulu-Natal Province, South Africa. S Afr Med J. 2020;0(0):13185. Epub 2020/12/19. PubMed PMID: 33334393.
140. Odinga MM, Kuria S, Muindi O, Mwakazi P, Njraini M, Melon M, et al. HIV testing amid COVID-19: community efforts to reach men who have sex with men in three Kenyan counties. Gates open research. 2020;4:117. Epub 2020/09/22. doi: 10.12688/gatesopenres.13152.1. PubMed PMID: 32954217; PubMed Central PMCID: PMCPMC7477340.
141. Darcis G, Vaira D, Moutschen M. Impact of coronavirus pandemic and containment measures on HIV diagnosis. Epidemiology and infection. 2020;148:e185. Epub 2020/08/25. doi: 10.1017/s0950268820001867. PubMed PMID: 32829742; PubMed Central PMCID: PMCPMC7463155.
142. Stanford KA, Friedman EE, Schmitt J, Spiegel T, Ridgway JP, Moore M, et al. Routine Screening for HIV in an Urban Emergency Department During the COVID-19 Pandemic. AIDS and behavior. 2020;24(10):2757-9. Epub 2020/05/04. doi: 10.1007/s10461-020-02899-x. PubMed PMID: 32361800; PubMed Central PMCID: PMCPMC7195825.
143. Chow EPF, Ong JJ, Denham I, Fairley CK. HIV testing and diagnoses during the COVID-19 pandemic in Melbourne, Australia. J Acquir Immune Defic Syndr. 2020;Publish Ahead of Print. Epub 2020/12/22. doi: 10.1097/QAI.0000000000002604. PubMed PMID: 33346567.

144. Maatouk I, Assi M, Jaspal R. Emerging impact of the COVID-19 outbreak on sexual health in Lebanon. Sex Transm Infect. 2020. Epub 2020/10/09. doi: 10.1136/sextrans-2020-054734. PubMed PMID: 33028650.
145. Stephenson R, Chavanduka TMD, Rosso MT, Sullivan SP, Pitter RA, Hunter AS, et al. Sex in the Time of COVID-19: Results of an Online Survey of Gay, Bisexual and Other Men Who Have Sex with Men's Experience of Sex and HIV Prevention During the US COVID-19 Epidemic. AIDS and behavior. 2020:1-9. Epub 2020/09/03. doi: 10.1007/s10461-020-03024-8. PubMed PMID: 32876905; PubMed Central PMCID: PMCPMC7464052.
146. Pampati S, Emrick K, Siegler AJ, Jones J. Changes in sexual behavior, PrEP adherence, and access to sexual health services due to the COVID-19 pandemic among a cohort of PrEP-using MSM in the South. medRxiv. 2020. Epub 2020/11/18. doi: 10.1101/2020.11.09.20228494. PubMed PMID: 33200145; PubMed Central PMCID: PMCPMC7668758.
147. Krakower D, Solleveld P, Levine K, Mayer KH. Impact of COVID-19 on HIV preexposure prophylaxis care at a Boston community health center. AIDS 2020 Conference, 2020.
148. Brawley S, Dinger J, Nguyen C, Anderson J. Impact of COVID-19 related shelterr-in-place orders on PrEP access, usage and HIV risk behaviours in the United States. AIDS 2020 Conference, 2020.
149. Dawson L, Kates J. Delivering HIV care and Prevention in the COVID Era: A National Survey of Ryan White Providers. KFF, 2020.
150. Chia CC, Chao CM, Lai CC. Diagnoses of syphilis and HIV infection during the COVID-19 pandemic in Taiwan. Sex Transm Infect. 2020. Epub 2020/10/22. doi: 10.1136/sextrans-2020-054802. PubMed PMID: 33082234.
151. Lourida G, Leonidou L, Psichogiou M, Chrysanthidis T, Protopappas K, Katsarolis I, et al. Effect of first three months of COVID pandemic on HIV services in Greece: a short survey among HIV physicians. Abstract Supplement HIV Glasgow - Virtual, 5-8 October, 2020, Journal of the International AIDS Society 2020;23:e25616.
152. Panagopoulos P, Petrakis V, Kyrgiannaki V, Kriaraki Z, Papazoglou D. Late presenters of HIV infection during COVID-19 pandemic. Abstract Supplement HIV Glasgow - Virtual, 5-8 October, 2020, Journal of the International AIDS Society. 2020;23:e25616.
153. Davey DLJ, Bekker LG, Mashele N, Gorbach P, Coates TJ, Myer L. PrEP retention and prescriptions for pregnant women during COVID-19 lockdown in South Africa. The lancet HIV. 2020. Epub 2020/08/08. doi: 10.1016/s2352-3018(20)30226-5. PubMed PMID: 32758479; PubMed Central PMCID: PMCPMC7398649.
154. Traeger MW, Patel P, Guy R, Hellard ME, Stoove MA, Australian Collaboration for Coordinated Enhanced Sentinel S. Changes in HIV preexposure prophylaxis prescribing in Australian clinical services following COVID-19 restrictions. AIDS. 2021;35(1):155-7. Epub 2020/12/05. doi: 10.1097/QAD.0000000000002703. PubMed PMID: 33273185.
155. Reyniers T, Rotsaert A, Thunissen E, Buffel V, Masquillier C, Van Landeghem E, et al. Reduced sexual contacts with non-steady partners and less PrEP use among MSM in Belgium during the first weeks of the COVID-19 lockdown: results of an online survey. Sex Transm Infect. 2020. Epub 2020/11/12. doi: 10.1136/sextrans-2020-054756. PubMed PMID: 33172917; PubMed Central PMCID: PMCPMC7656903.
156. Chow EPF, Hocking JS, Ong JJ, Schmidt T, Buchanan A, Rodriguez E, et al. Changing the Use of HIV Pre-exposure Prophylaxis Among Men Who Have Sex With Men During the COVID-19 Pandemic in Melbourne, Australia. Open forum infectious diseases. 2020;7(7):ofaa275. Epub 2020/07/25. doi: 10.1093/ofid/ofaa275. PubMed PMID: 32704518; PubMed Central PMCID: PMCPMC7337741.
157. Hammoud MA, Grulich A, Holt M, Maher L, Murphy D, Jin F, et al. Substantial Decline in Use of HIV Preexposure Prophylaxis Following Introduction of COVID-19 Physical Distancing Restrictions in Australia: Results From a Prospective Observational Study of Gay and Bisexual Men. J Acquir Immune Defic Syndr. 2021;86(1):22-30. Epub 2020/10/08. doi:

10.1097/QAI.0000000000002514. PubMed PMID: 33027151; PubMed Central PMCID: PMCPMC7727320.
158. Junejo M, Girometti N, McOwan A, Whitlock G. HIV postexposure prophylaxis during COVID-19. The lancet HIV. 2020;7(7):e460. Epub 2020/05/29. doi: 10.1016/s2352-3018(20)30146-6. PubMed PMID: 32464106; PubMed Central PMCID: PMCPMC7247797.
159. Sánchez-Rubio J, Vélez-Díaz-Pallarés M, Rodríguez González C, Sanmartin Fenollera P, García Yubero C, Fernández-Pacheco García-Valdecasas M. HIV postexposure prophylaxis during the COVID-19 pandemic: experience from Madrid. Sex Transm Infect. 2020. Epub 2020/07/19. doi: 10.1136/sextrans-2020-054680. PubMed PMID: 32680842.
160. Chow EPF, Hocking JS, Ong JJ, Phillips TR, Fairley CK. Postexposure prophylaxis during COVID-19 lockdown in Melbourne, Australia. The lancet HIV. 2020;7(8):e528-e9. Epub 2020/07/21. doi: 10.1016/s2352-3018(20)30204-6. PubMed PMID: 32687796; PubMed Central PMCID: PMCPMC7367677.
161. Siedner MJ, Kraemer JD, Meyer MJ, Harling G, Mngomezulu T, Gabela P, et al. Access to primary healthcare during lockdown measures for COVID-19 in rural South Africa: an interrupted time series analysis. BMJ Open. 2020;10(10):e043763. Epub 2020/10/07. doi: 10.1136/bmjopen-2020-043763. PubMed PMID: 33020109; PubMed Central PMCID: PMCPMC7536636.
162. Qiao S, Yang X, Sun S, Li X, Mi T, Zhou Y, et al. Challenges to HIV service delivery and the impacts on patient care during COVID-19: perspective of HIV care providers in Guangxi, China. AIDS Care. 2020:1-7. Epub 2020/11/28. doi: 10.1080/09540121.2020.1849532. PubMed PMID: 33242981.
163. Qiao S, Li Z, Weissman S, Li X, Olatosi B, Davis C, et al. Disparity in HIV Service Interruption in the Outbreak of COVID-19 in South Carolina. AIDS and behavior. 2020:1-9. Epub 2020/08/29. doi: 10.1007/s10461-020-03013-x. PubMed PMID: 32856176; PubMed Central PMCID: PMCPMC7453068.
164. Kowalska JD, Skrzat-Klapaczyńska A, Bursa D, Balayan T, Begovac J, Chkhartishvili N, et al. HIV care in times of the COVID-19 crisis - Where are we now in Central and Eastern Europe? International journal of infectious diseases : IJID : official publication of the International Society for Infectious Diseases. 2020;96:311-4. Epub 2020/05/16. doi: 10.1016/j.ijid.2020.05.013. PubMed PMID: 32413608; PubMed Central PMCID: PMCPMC7211569.
165. COVID-19 Situation Report #35, Reporting period 9 December - 22 December 2020. The Global Fund.
166. Lagat H, Sharma M, Kariithi E, Otieno G, Katz D, Masyuko S, et al. Impact of the COVID-19 Pandemic on HIV Testing and Assisted Partner Notification Services, Western Kenya. AIDS and behavior. 2020;24(11):3010-3. Epub 2020/06/04. doi: 10.1007/s10461-020-02938-7. PubMed PMID: 32488552; PubMed Central PMCID: PMCPMC7265868.
167. Fourth annual progress report of the hIV Prevention 2020 Road Map implementation. Geneva: UNAIDS, 2020.
168. Jewell BL, Mudimu E, Stover J, Ten Brink D, Phillips AN, Smith JA, et al. Potential effects of disruption to HIV programmes in sub-Saharan Africa caused by COVID-19: results from multiple mathematical models. The lancet HIV. 2020;7(9):e629-e40. Epub 2020/08/11. doi: 10.1016/s2352-3018(20)30211-3. PubMed PMID: 32771089.
169. Mitchell KM, Dimitrov D, Silhol R, Geidelberg L, Moore M, Liu A, et al. Estimating the potential impact of COVID-19-related disruptions on HIV incidence and mortality among men who have sex with men in the United States: a modelling study. medRxiv. 2020. Epub 2020/11/12. doi: 10.1101/2020.10.30.20222893. PubMed PMID: 33173893; PubMed Central PMCID: PMCPMC7654885.
170. Jenness SM, Le Guillou A, Chandra C, Mann LM, Sanchez T, Westreich D, et al. Projected HIV and Bacterial STI Incidence Following COVID-Related Sexual Distancing and Clinical Service Interruption. medRxiv. 2020. Epub 2020/10/08. doi: 10.1101/2020.09.30.20204529. PubMed PMID: 33024979; PubMed Central PMCID: PMCPMC7536881.

171. Booton RD, Fu G, MacGregor L, Li J, Ong JJ, Tucker JD, et al. Estimating the impact of disruptions due to COVID-19 on HIV transmission and control among men who have sex with men in China. medRxiv. 2020. Epub 2020/10/22. doi: 10.1101/2020.10.08.20209072. PubMed PMID: 33083811; PubMed Central PMCID: PMCPMC7574267.
